# A Nanostructured Ru‐Mn‐Nb Alloy with Oxygen‐Enriched Boundaries for Ampere‐Level Hydrogen Evolution

**DOI:** 10.1002/advs.202501976

**Published:** 2025-04-26

**Authors:** Jie Li, Xue Wang, Jun Yu, Kai Xu, Zhe Jia, Hongkun Li, Lei Ren, Yiyuan Yang, Keke Chang, Yangyang Li, Xiangfa Liu, Jian Lu, Sida Liu

**Affiliations:** ^1^ Key Laboratory for Liquid‐Solid Structural Evolution & Processing of Materials Ministry of Education Shandong University Jinan 250061 China; ^2^ Key Laboratory of Advanced Marine Materials Ningbo Institute of Materials Technology and Engineering Chinese Academy of Sciences Ningbo Zhejiang 315201 China; ^3^ Laboratory for Multiscale Mechanics and Medical Science SV LAB School of Aerospace Xi'an Jiaotong University Xi'an 710049 China; ^4^ School of Materials Science and Engineering Jiangsu Key Laboratory for Advanced Metallic Materials Southeast University Nanjing 211189 China; ^5^ Hong Kong Branch of National Precious Metals Material Engineering Research Center City University of Hong Kong Hong Kong SAR China

**Keywords:** crystal‐crystal heterostructure, electrocatalysis, hydrogen evolution reaction, magnetron sputtering, medium entropy alloy

## Abstract

Development of active and cost‐effective electrocatalysts to substitute platinum‐based catalysts in alkaline hydrogen evolution reactions (HERs) remains a challenge. The synergistic effect between different elements in alloy catalysts can regulate electronic structure and thereby provide an abundance of catalytic sites for reactions. Thus, alloy catalysts are suitable candidates for future energy applications. Conventional methods for enhancing the performance of alloy catalysts have mainly focused on element composition and thus have often neglected to examine catalyst design. In this paper, a ruthenium–manganese–niobium alloy catalyst (Ru_62_Mn12Nb_21_O_5_) is reported with a supra‐nanocrystalline dual‐phase structure that is fabricated through combinatorial magnetron co‐sputtering at ambient temperatures. The induced crystal–crystal heterostructure of Ru_62_Mn_12_Nb_21_O_5_ reduced system energy, thereby achieving balance between stability and catalytic activity. Ru_62_Mn_12_Nb_21_O_5_ exhibited excellent HER performance, as demonstrated by low HER overpotential (18 mV at 10 mA cm^−2^) and robust stability (300 h at 1.2 A cm^−2^). Moreover, oxygen‐rich interfaces in Ru_62_Mn_12_Nb_21_O_5_ enhanced charge transfer and the kinetics of water dissociation as well as optimized hydrogen adsorption/desorption processes, thus boosting HER performance. The crystal–crystal heterostructure and oxygen‐rich interfaces in Ru_62_Mn_12_Nb_21_O_5_ are induced by its dual‐phase nanocrystalline structure, which represents a new structural design for enhancing the performance of catalysts for sustainable energy development.

## Graphical abstract

Ru_62_Mn_12_Nb_21_O_5_ medium‐entropy alloy with a crystal‐crystal dual‐phase structure is deposited on a carbon cloth substrate by the magnetron sputtering method. The oxygen‐rich interface induced by the rich Mn phase and the poor Mn phase optimized the electronic structure of the catalyst, adjusted the adsorption and desorption processes of intermediates, and thus achieved promising hydrogen evolution activity and high current stability.

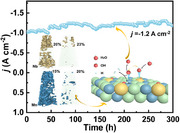

## 1 Introduction

The widespread use of fossil fuels has made for significant pollution and development challenges, drawing global interest.^[^ ^1^, ^2^, ^3^ ^]^ According to the International Energy Agency, carbon dioxide emissions are expected to reach 35.7 Gt year^−1^ in 2040, due to global energy demand increasing by 30%.^[^ ^4^ ^]^ A promising solution to this energy crisis and associated environmental pollution is hydrogen (H_2_), as it emits no carbon (C) compounds when combusted and has a high maximum energy density (142 MJ kg^−1^).^[^ ^5^ ^]^ Among the various hydrogen energy production techniques, electrochemical water (H_2_O) splitting is considered an effective, sustainable, and environmentally friendly method.^[^ ^6^ ^]^ The electrochemical H_2_ evolution reaction (HER) is a critical half‐reaction in H_2_O splitting that provides a practical strategy for transforming electrical energy into chemical energy. However, HER efficiency is limited in alkaline electrolytes, due to an increased energy barrier to H_2_O dissociation.^[^ ^7^, ^8^ ^]^ Thus, there remains a need to develop advanced electrocatalysts with high capacities for H_2_O dissociation in alkaline electrolytes.

Currently, catalysts based on precious metals, platinum (Pt) and ruthenium (Ru), are the most effective for electrochemical H_2_O splitting, due to their high exchange‐current densities and low Tafel slopes.^[^ ^9^, ^10^ ^]^ Pt‐based catalysts are the most widely used, but as Pt is expensive and lacks sufficient stability, their potential applications are limited. A theoretically viable alternative to Pt‐based catalysts are Ru‐based catalysts, as they exhibit excellent H_2_O‐dissociation abilities and the cost of Ru is ≈5% that of Pt.^[^ ^11^, ^12^ ^]^ However, the industrial application of Ru‐based catalysts is challenging, as the strong interaction between hydrogen and ruthenium atoms can impede H_2_ desorption and reduce the H_2_ production rate.^[^ ^13^, ^14^, ^15^ ^]^ Therefore, it is critical to rationally tune the electronic structures and desorption energies of Ru‐based catalysts. One promising strategy for such tuning involves constructing heterostructures,^[^ ^16^, ^17^, ^18^ ^]^ as this creates a heterogeneous phase with different electronegativities and work functions. Consequently, electrons undergo directional transfer at the heterogeneous phase interface. This transfer induces charge redistribution in the active center and optimizes its electronic structure and charge transport characteristics, leading to enhanced catalytic activity.^[^ ^19^ ^]^ Recently, numerous studies have revealed that Ru‐based heterostructures, such as Pt–Ru/RuO_2_,^[^ ^20^ ^]^ copper‐doped Ru/RuSe_2_,^[^ ^21^ ^]^ and SA–Ru–MoS_2_ (where SA = single atom),^[^ ^22^ ^]^ exhibit excellent catalytic performance in electrochemical H_2_O splitting due to their metals’ synergistic effects. However, challenges remain with the application of Ru‐based heterostructure catalysts, such as maintaining catalyst morphology and support interactions over prolonged periods to prevent a loss of catalytic activity. In addition, the complex synthesis procedures of Ru‐based heterostructure catalysts hinder their industrial‐scale production.

One‐step design and fabrication approaches offer a promising solution to the above‐mentioned problems and may allow development of the next generation of heterostructure catalysts. For example, a crystal glass‐nanostructured catalyst comprising AlMnRu was synthesized based on phase diagram calculations and using combined magnetron co‐sputtering. AlMnRu showed excellent catalytic performance in the HER, but its glass phase tended to crystallize after electrochemical reactions and thus decrease its stability.^[^ ^23^ ^]^ To solve this problem and achieve a balance between stability and catalytic activity, two approaches have been proposed. The first involves introducing multiple components to enhance entropy stabilization effects.^[^ ^24^, ^25^ ^]^ However, this requires careful selection of components to avoid adding inactive materials that could reduce catalytic efficiency. The second approach involves inducing the formation of crystal–crystal structures to reduce system energy, allowing partial replacement of a catalyst's structure. This structural modification enables the formation of grain boundaries, which can act as active sites.^[^ ^26^, ^27^ ^]^ Thus, this approach is most suitable for catalyst preparation.

In this study, we synthesized a RuMnNbO medium‐entropy alloy (MEA) film using a magnetron sputtering technique and examined its structure and catalytic activity. The 3D atom probe tomography (3D‐APT) and transmission electron microscopy (TEM) results indicated RuMnNbO MEA film was composed of a Mn‐rich/Mn‐poor dual phase, which induced the formation of O‐rich interfaces. The phase interface is formed between the Mn‐rich and Mn‐poor phases. Compared to the relatively stable Mn‐rich and Mn‐poor phases, the energy at the phase interface is usually relatively high. From a thermodynamic perspective, oxygen tends to accumulate at the interface, becoming oxygen‐rich interfaces, thereby reducing the overall energy.^[^ ^28^ ^]^ Because of the tiny size (1.38 nm) of the dual phase and the high density of the O‐rich interfaces, RuMnNbO was a supra‐nano solid solution that exhibited exceptional catalytic performance in electrochemical H_2_ evolution, as evidenced by overpotentials of only 18 and 64.6 mV for 10 and 100 mA cm^−2^, respectively. Moreover, RuMnNbO had a Tafel slope of 24.3 mV dec^−1^, a mass activity of 81.2 mA mg^−1^, as well as turnover frequency (TOF) of 34.8 s^−1^. We employed density functional theory (DFT) calculations to systematically study the structure–property relationships of RuMnNbO. The results revealed that the presence of O atoms in crystal–crystal interfaces reduced the energy barrier in which active hydrogen (*H) was transformed into H_2_. In addition, these interfacial O atoms increased the efficiency of the H_2_O dissociation step, resulting in RuMnNbO being superior to a Pt‐based catalyst for electrochemical H_2_ evolution. Subsequently, it was found that RuMnNbO exhibited excellent electrochemical stability, i.e., 1.2 A cm^−2^ for at least 300 h, and its H_2_ production cost could be reduced to $0.67 kg^−1^ H_2_. Therefore, RuMnNbO is a dual‐crystalline‐phase nanostructured catalyst with a novel basis for its balance of stability and catalytic activity, which paves the path for the development of new‐generation catalysts.

## 2 Results and Discussion

**Figure 1:**
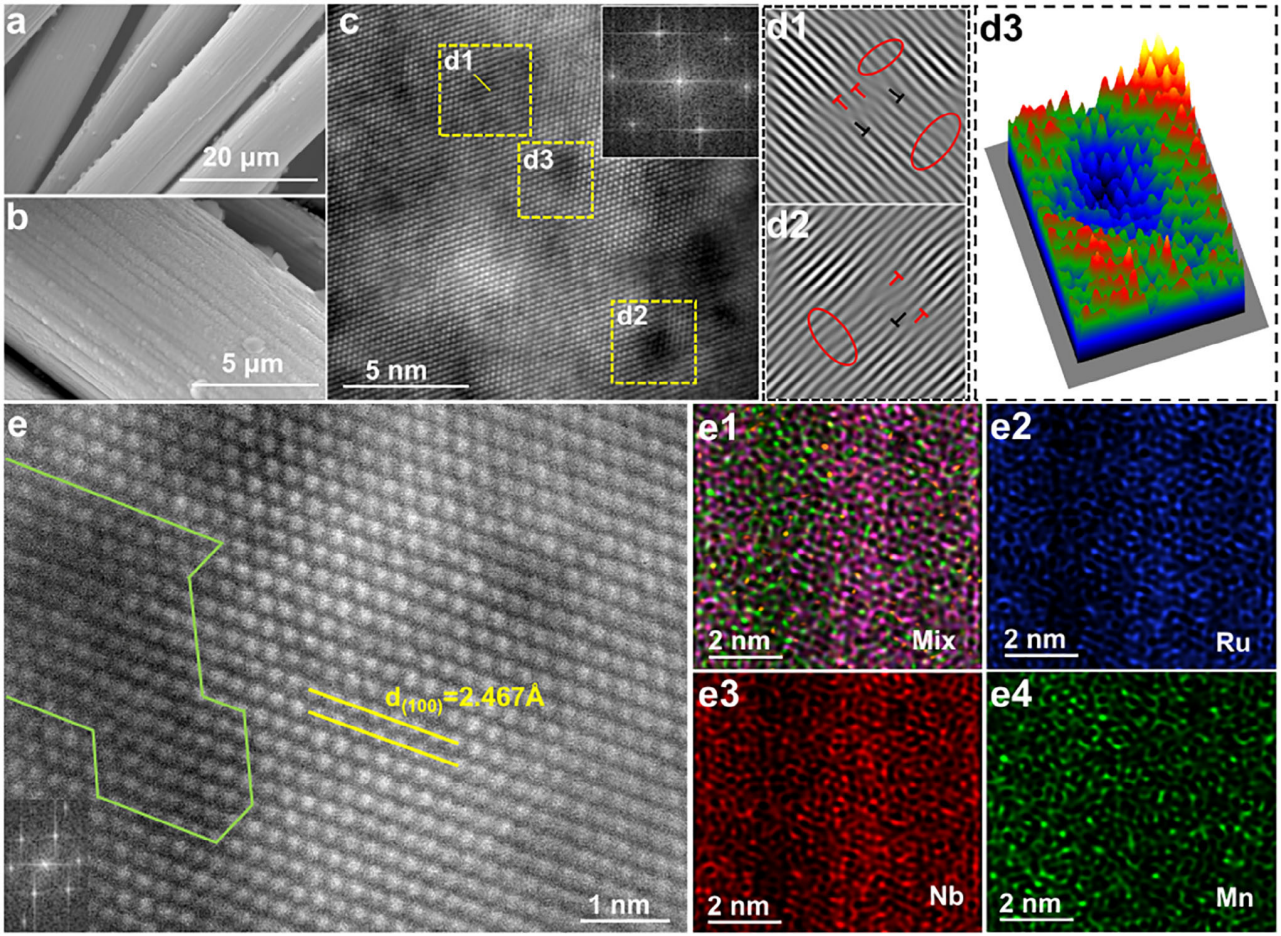
Microstructural characterization of M‐MEA film. a, b) SEM images and c) High‐resolution TEM image of as‐deposited M‐MEA film. d1‐2) Inverse fast Fourier transform images of the area surrounded by the yellow square in (c). d3) 3D atom‐overlapping Gaussian function fitting mapping of the aforementioned area in (c). e‐e4) AC‐HAADF‐STEM image and corresponding elemental mappings of M‐MEA film.

MEA film was prepared via a simple physical vapor deposition method. Specifically, elemental Ru, Mn, and Nb were deposited on a carbon cloth through co‐sputtering under an argon atmosphere to form a medium‐Mn‐concentration MEA (M‐MEA) film (Figure S1, Supporting Information). The surface of the carbon cloth substrates was completely coated by the M‐MEA film, which had a thickness of ≈750 nm. High‐magnification scanning electron microscopy (SEM) revealed that M‐MEA film had a rough surface, which accounted for the large specific exposed area (**Figure** **1**a,b; Figure S1c–f, Supporting Information). Inductively coupled plasma–optical emission spectrometry (ICP‐OES) (Figure S2, Supporting Information) revealed that the M‐MEA film had a Ru‐to‐Nb‐to‐Mn mass ratio of 71.5:20.3:8.2, and a Ru‐to‐Nb‐to‐Mn mole ratio of 65.8:20.3:13.9, which is in line with the results obtained from energy‐dispersive spectroscopy (EDS) (Figure S3, Supporting Information). TEM was used to perform EDS mapping (Figure S3c, Supporting Information) of the M‐MEA film and indicated that its elemental distributions of Ru, Mn, and Nb almost completely overlapped. This result confirmed that there was a reasonably homogeneous macro‐level distribution of each element in the M‐MEA film catalyst. The X‐ray diffractometry (XRD) pattern of the M‐MEA film (The illustration in Figure S3a, Supporting Information) indicated that it mainly consisted of hexagonal Ru (PDF#06‐0663). The broadening and negative shift of the diffraction peaks are attributed to the ultra‐small crystal size and lattice expansion triggered by the multiple transition metals in the M‐MEA film. A selected‐area electron diffraction (SAED) image showed polycrystalline diffraction with clear (002) crystal planes, confirming the polycrystalline structure of the MEA film (Figure S4a,b, Supporting Information). Aberration‐corrected high‐angle annular dark‐field scanning transmission electron microscopy (AC‐HAADF‐STEM) was employed to accurately characterize the morphology and structural details of the M‐MEA film. Inspired by the relationship between contrast and proton number, the contrast fluctuation in Figure 1c directly reflected the variation in the elemental concentration. This result evidenced that M‐MEA comprised two phases, in which the size of the dark phase was ≈1.38 nm (Figures S4c,d, Supporting Information). 3D atom‐overlapping Gaussian function fitting mapping was used to visualize the co‐existence of the two phases, as shown in Figure 1d3. Due to the rapid cooling rate and differences in atomic radii, an enlarged lattice parameter was recorded, and the transient dislocations and twisting caused by lattice mismatch at the interface of the two phases persisted^[^ ^29^, ^30^ ^]^ (Figure 1d1–d2). To determine the composition of the two phases, atomic elemental distribution mapping was conducted based on Figure 1e. According to Figure 1e1–e4, a higher concentration of Mn appeared to be present in the dark region than in the light region. Moreover, the line scanning results in Figure S4e,f (Supporting Information) demonstrated that as the arrow crossed the interface, i.e., from the light region to the dark region, the concentration of Mn increased and the concentration of Ru decreased. In addition to the metallic components, a minimal distribution of O was observed at the interface, which is discussed later. Based on these results, high‐Mn‐MEA (H‐MEA) film and low‐Mn‐MEA (L‐MEA) film were used to distinguish these two sections of M‐MEA film according to their concentrations of Mn. To compare their individual performances and synergistic effects, H‐MEA film and L‐MEA film were fabricated using the same process, as shown in Figure S5 (Supporting Information). Furthermore, the adhesion behavior of bubbles and water at self‐supporting electrodes was investigated by the contact angle testing. As displayed in Figure S6a–c (Supporting Information), M‐MEA film exhibits larger contact angles than comparative samples, indicating a weaker adhesion of bubbles on the M‐MEA film than that on the RuNb and RuMn films. Besides, M‐MEA films exhibit enhanced water affinity compared to RuNb and RuMn films (Figure S6d‐f1, Supporting Information), as evidenced by their hydrophilic characters with small water contact angles.

**Figure 2:**
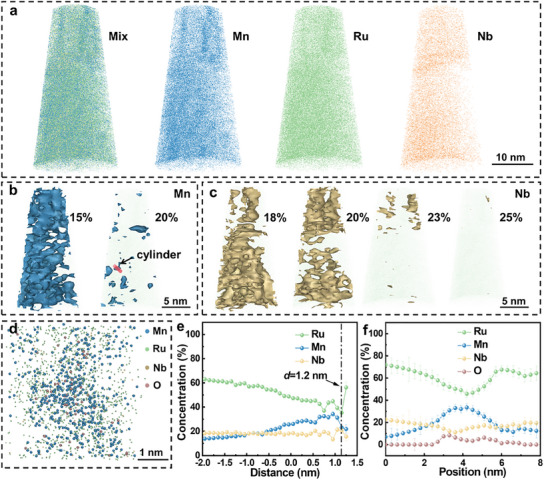
3D heterogeneity of the M‐MEA film. a) 3D reconstruction of a 3D‐APT dataset, showing the elemental distribution and multicomponent of the M‐MEA film. b) Iso‐composition surface with 15 at% and 20 at% Mn concentration thresholds (where at% = atomic percentage). c) Iso‐composition surface with 18 at% and 25 at% Nb concentration thresholds. d) Plane‐view slice from the 3D‐APT dataset in (b), revealing Mn‐enriched regions. e) Compositional proxigram generated using the 20% Mn iso‐composition surface. f) Atom distribution showing the composition change across the cylinder in (b). The error bars are standard deviations.

To further characterize the phase distribution of M‐MEA film, 3D‐APT was employed (**Figure** **2**). The atomic reconstruction showed that at the microscale in M‐MEA film, the distribution of Mn and Ru was heterogeneous but that of Nb was rather homogeneous (Figure 2a). This difference was visualized by constructing 3D contour plots of Mn and Nb at different concentration thresholds (Figure 2b,c). With a threshold concentration of 15 at%, a large proportion of the matrix was covered by blue iso‐concentration surfaces. Even when the threshold concentration was increased to 20 at%, a certain proportion of the matrix remained covered by blue iso‐concentration surfaces. In contrast to the average concentration of 13 at%, these results reflected the dominant presence of Mn‐rich and Mn‐poor regions, which is consistent with Figure 1. Moreover, the plane‐view slice (Figure 2d) from Figure 2b showed that the concentration of Mn decreased quickly around the Mn‐rich region, indicating that Mn‐rich and Mn‐poor regions were distributed adjacent to each other. The average concentration of Nb was 22 at%, and the iso‐concentration surfaces of Nb at different threshold concentrations confirmed the homogeneous distribution of Nb. Subsequently, the average dimension of the Mn‐rich phases was calculated. The compositional proximity histogram (proxigram) generated using the 20 at% Mn iso‐composition surface (Figure 2e) indicated that the size of the Mn‐rich phase was ≈1.2 nm, consistent with the results of TEM. The composition of cylinder in Figure 2b was statistically analyzed, and its composition profile (Figure 2f) confirmed the spatial distribution differences between elemental Ru and Mn. In summary, based on the 3D APT and TEM results, it was determined that M‐MEA film consisted of an Mn‐poor matrix interspersed with nano‐sized Mn‐rich phases, which averaged 1.3 nm in size. In addition, O was primarily localized at the interface between Mn‐rich phases and the Mn‐poor matrix.

**Figure 3:**
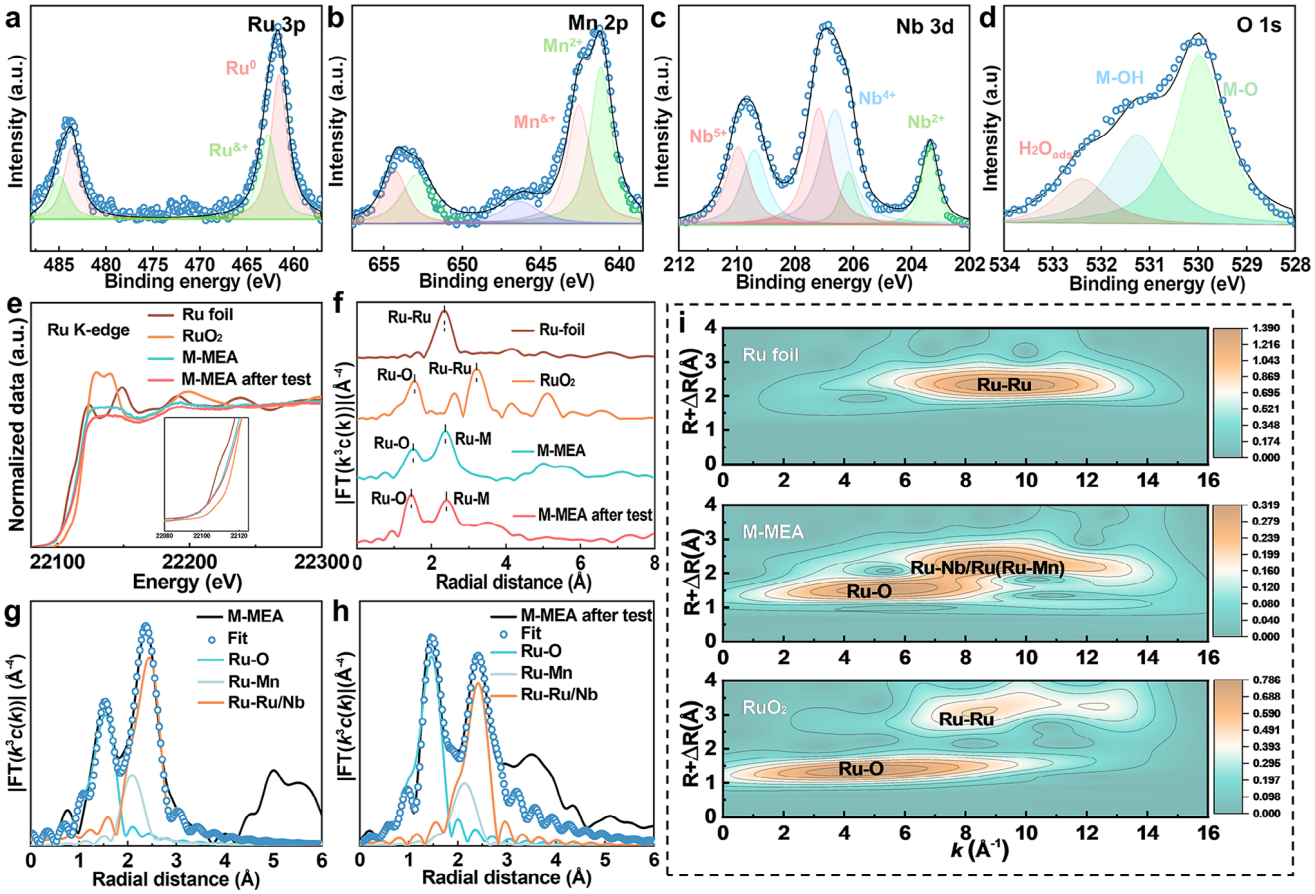
Data from electronic structural investigations of the M‐MEA film. XPS spectra of the a) Ru 3p, b) Mn 2p, c) Nb 3d, and d) O 1s regions of fresh M‐MEA film. e) Ru K‐edge XANES spectra of fresh M‐MEA film, M‐MEA film after stability testing, Ru foil, and RuO_2_; the inset is the absorption edge. f) Fourier‐transformed Ru K‐edge EXAFS spectra of fresh and stability‐tested M‐MEA film and standard samples (Ru foil and RuO_2_). Fitting result of Fourier‐transformed Ru K‐edge EXAFS spectra of fresh M‐MEA film g) and stability‐tested M‐MEA film h). i) Wavelet transform for the k^3^‐weighted EXAFS Ru K‐edge signals of Ru foil, RuO_2_, and fresh M‐MEA film.

The electronic structure of the M‐MEA film played an important role in its electrochemical activity and thus was measured by X‐ray photoelectron spectroscopy (XPS). After calibration with C 1s with the binding energy of 284.8 eV, and the full XPS spectrum of the M‐MEA film was obtained (Figure S7, Supporting Information). It demonstrated that elemental Ru, Mn, Nb, and O were present on the surface of the M‐MEA film, with no impurities apparent, consistent with the results of EDS. As shown in **Figure** **3**a, the high‐resolution Ru 3p spectra of the M‐MEA film could be deconvoluted into two parts, corresponding to metallic Ru^0^ and Ru^&+^ species, at 461.6/483.6 and 462.7/484.8 eV, respectively. Compared with the Ru 3p spectra of binary films (RuNb, RuMn films) (Figure S8, Supporting Information), the Ru 3p spectrum of the M‐MEA film exhibited a downward shift, implying that it had a greater electron density on Ru. Due to differences in electronegativity, the d–d electron transfer and p–π orbital coupling of Ru, Mn, Nb, and O in the M‐MEA film induced the generation of low‐valence Ru sites, leading to an optimized local charge environment.^[^ ^31^, ^32^ ^]^ In contrast, positive states were dominant in the Mn 2p and Nb 3d spectra (Figure 3b,c), possibly induced by the aforementioned electron transfer and oxidation. This observation was supported by the signal for metal–oxygen bonding states (Figure 3d).

Subsequently, X‐ray absorption near‐edge spectroscopy (XANES) and extended X‐ray absorption fine structure (EXAFS) spectroscopy were conducted to further clarify the electronic structures, coordination states of Ru species in M‐MEA film. As shown in Figure 3e, the absorption edge in the Ru K‐edge XANES spectra of the M‐MEA film was between the absorption edge of Ru foil and that of RuO_2_. This result reveals oxidation state of Ru species was between 0 and +4, demonstrating that electron transfer occurred between the elements in the M‐MEA film, as mentioned in the discussion on XPS results. Following fitting and calculation, the valence state of Ru was ≈+1.3 on the surface of as‐prepared M‐MEA film (Figure S9 and Table S2, Supporting Information). In addition, the post‐edges in the M‐MEA film exhibited deviations both in intensity and shape compared with their foil and oxide counterparts, indicating that there was orbital hybridization of elements in the M‐MEA film.^[^ ^33^ ^]^ The EXAFS spectrum of the M‐MEA film (Figure 3f) contained two distinct scattering peaks, ≈2.055 and 2.600 Å, which were associated with Ru–O and metallic Ru–M (M = Ru, Mn, and Nb) species, respectively. These results suggested that O‐coordinated Ru and metal‐coordinated Ru co‐existed in the M‐MEA film. To reveal the detailed coordination environment of Ru atoms in M‐MEA film, the EXAFS curve of Ru in *R* space was fitted, shown in Figure 3g and Table S3 (Supporting Information). The fitting results and wavelet transform (Figure 3i) of the Ru K‐edge EXAFS spectrum indicated that the M‐MEA film had an average Ru–O bond length of 2.055 Å with a coordination number of 3.7. In addition, the Ru–M signal of the M‐MEA film could be deconvoluted into two parts: an Ru–Mn bond with an average bond length of 2.513 Å and average coordination number of 2.8, and an Ru–Ru/Nb bond with an average bond length of 2.712 Å and average coordination number of 8.6. These findings align with the TEM and XRD results. The wider distributions along the *k* axis in the M‐MEA film compared with those along the *k* axis in their standard foils and oxides were due to the complex coordination environment of M‐MEA film, shown in Figure S10 (Supporting Information). Detailed structural information is summarized in Table S3 (Supporting Information).

**Figure 4:**
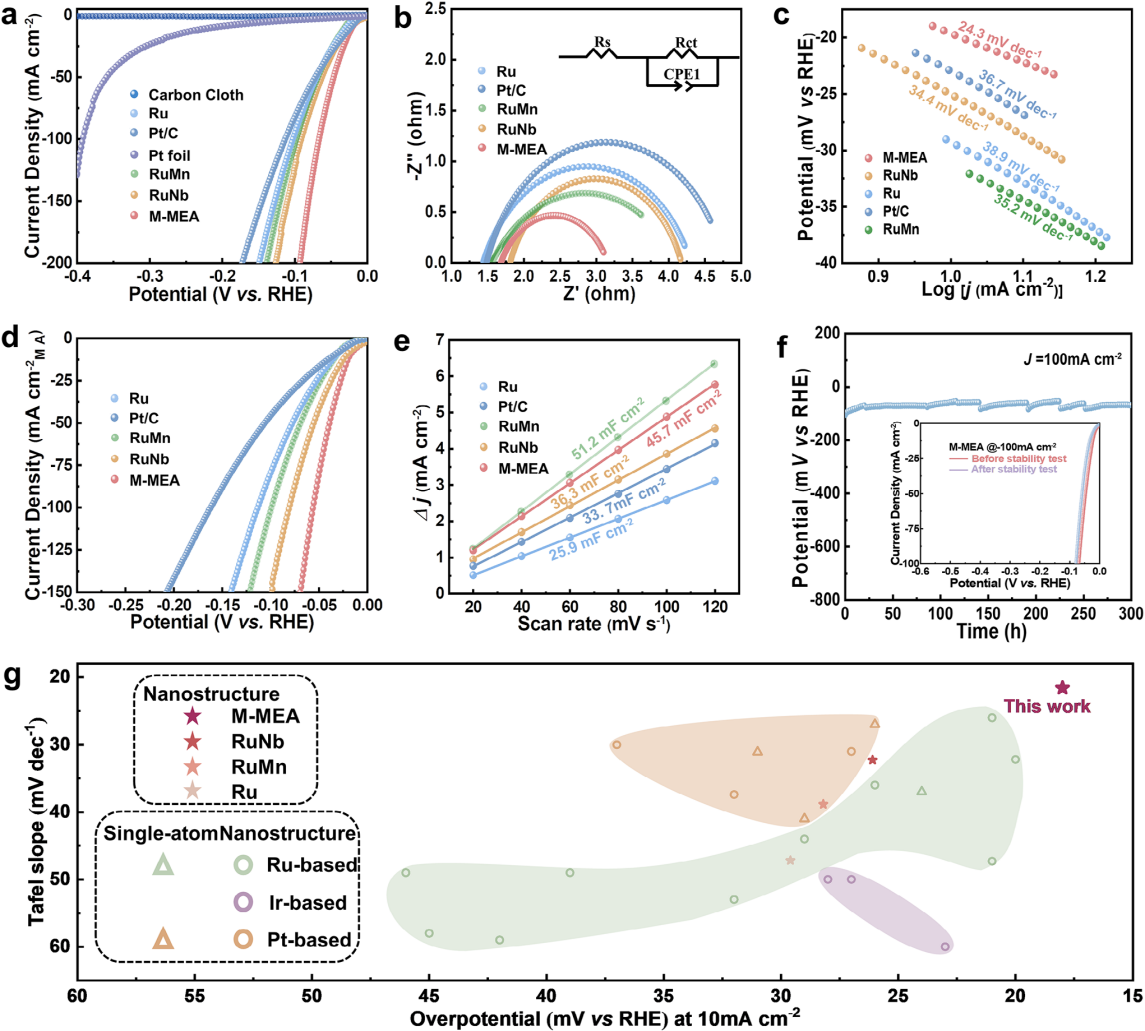
Electrocatalytic performance of as‐received catalysts in 1 M KOH solution. a) LSV curves at a scan rate of 5 mV s^−1^ (with iR compensation). b) Nyquist plots of as‐received catalysts. c) Tafel slopes. d) Mass activities of films and Pt/C in 1.0 M KOH. e) Capacitive current plots of films and Pt/C. f) Stability test of M‐MEA film in the HER in 1 M KOH solution at 100 mA cm^−2^. The inset shows the LSV curves before and after the stability test (scanning at 5 mV s^−1^). g) HER catalytic performances of M‐MEA film and previously reported noble metal‐based catalysts.

Fresh crystal–crystal nanostructured M‐MEA film exhibited excellent HER activity in a 1 mol L^−1^ potassium hydroxide (KOH) electrolyte. For comparison, commercial Pt/C, Pt foil, Ru film, H‐MEA film, L‐MEA film, and binary film with the same metal element ratio as the M‐MEA film (Ru_75_Nb_25_ labelled as RuNb, Ru_83_Mn_17_ labelled as RuMn) were also assessed at the same conditions. The cyclic voltammetry (CV) curves obtained in H_2_‐saturated alkaline electrolyte were used to calibrate all measured potentials to reversible hydrogen electrode (RHE) (Figure S11a, Supporting Information). The resulting polarization linear sweep voltammetry (LSV) curves are shown in **Figure** **4**a and Figure S11b (Supporting Information). For a current density of 10 mA cm^−2^, the overpotential for M‐MEA film (18 mV) was lower than the overpotential required by the RuNb film (26.1 mV), RuMn film (30.4 mV), Ru film (29.6 mV), commercial Pt/C (23.4 mV), Pt foil (213 mV), H‐MEA film (25.3 mV), and L‐MEA film (20.3 mV) (Table S4, Supporting Information). Moreover, the M‐MEA film demonstrated excellent high‐current‐density activity, with only 64.6 mV required to reach 100 mA cm^−2^. Electrochemical impedance spectroscopy (EIS) was used to study the catalytic kinetics on the electrochemical interfaces (Figure 4b; Figure S11c, Supporting Information). Compared with the charge transfer resistance (*Rct*) of RuNb film (2.36 Ω), RuMn film (2.56 Ω), Ru film (2.87 Ω), commercial Pt/C (3.30 Ω), H‐MEA film (2.05 Ω), and L‐MEA film (1.96 Ω), that of the M‐MEA film was smaller (1.48 Ω). This result implied that the M‐MEA film had the lowest charge transfer resistance and fastest charge transfer rate for the HER reaction.^[^ ^34^ ^]^ Furthermore, the excellent HER activity of the M‐MEA film was reflected on the smallest Tafel slope, i.e., 24.3 mV dec^−1^ (Figure 4c; Figure S11d, Supporting Information), which accelerated the slow chemical kinetics step.^[^ ^35^ ^]^ This performance exhibits among the best of representative state‐of‐the‐art noble HER electrocatalysts^[^ ^36^, ^37^, ^38^, ^39^, ^40^, ^41^, ^42^, ^43^, ^44^, ^45^, ^46^, ^47^, ^48^, ^49^, ^50^, ^51^, ^52^, ^53^, ^54^, ^55^ ^]^ (Figure 4g; Table S5, Supporting Information). However, the HER performance of the M‐MEA film was poor under acidic conditions, as evidenced by an overpotential at 10 mA cm^−2^ of 48.3 mV and a Tafel slope of 63.5 mV dec^−1^ (Figure S12, Supporting Information). This poor performance was possibly caused by the reduction of reactive sites or change in reaction routes (Figure S12d–f, Supporting Information) under acidic conditions.

In addition, the mass activities of the samples were investigated by standardizing the current density and the weights of the precious metals in these catalysts. As shown in Figure 4d, the M‐MEA film was 81.2 mA mg^−1^ at an overpotential of 50 mV (versus RHE), ≈5.2 times than the Pt/C, i.e., 15.6 mA mg^−1^. In addition, the intrinsic catalytic activities of the samples were evaluated by their double‐layer capacitances (*C_dl_*). The CV curves at different scanning speeds revealed that compared with Pt/C, the M‐MEA film had a larger electrochemical active surface area (Figure 4e; Figures S11e–h and S13, Supporting Information), indicating that it contained more active sites, which increased the efficiency of the HER. Furthermore, to reveal the catalytic ability of single active site, the TOFs of M‐MEA film, commercial Pt/C were calculated.^[^ ^56^ ^]^ Given Cu(II)‐ion underpotential deposition,^[^ ^57^, ^58^ ^]^ the TOF of the M‐MEA film was ≈0.6*|j|* and Pt/C was ≈0.11*|j|*. Therefore, at 50 mV (versus RHE), the M‐MEA film achieved a TOF of 34.8 s^−1^, exceeding that of Pt/C, i.e., 3.4 s^−1^, and those of recently reported precious metal‐based HER catalysts^[^ ^23^, ^52^, ^58^, ^59^, ^60^, ^61^, ^62^, ^63^, ^64^, ^65^, ^66^ ^]^ (Figure S14 and Table S6, Supporting Information).

Long‐term stability is another crucial factor affecting the electrochemical performance for electrocatalysts, especially industrial applications. The LSV curve of the M‐MEA film after a 300 h stability test at −100 mA cm^−2^ (Figure 4f) showed that its overpotential was not significantly amplified, demonstrating its long‐term stability. A continuous cycling test at a potential range of 0 to −0.7 V further highlighted the stability of M‐MEA film (Figure S15a, Supporting Information). Specifically, it demonstrated a negligible increase in overpotential after 25 000 cycles at 80 mA cm^−2^. Taken together, the above‐mentioned results demonstrate that the M‐MEA film possessed excellent durability during the HER process under its efficient utilization of active sites and morphology favoring mass and electron transport. Next, a high‐current stability test was carried out to evaluate the sustainability of M‐MEA film for large‐scale H_2_ production (Figure S15b,d, Supporting Information). The results showed that the M‐MEA film could operate steadily for 300 h at 1.2 mA cm^−2^ in a three‐electrode cell, representing exceptional electrochemical H_2_ production stability compared with other noble metal‐based catalysts.^[^ ^7^, ^31^, ^40^, ^41^, ^48^, ^51^, ^54^, ^63^, ^67^, ^68^, ^69^, ^70^, ^71^, ^72^, ^73^ ^]^ ICP‐OES investigations revealed the concentration of Ru leached from film remained stable, even during high‐current experiments. This indicated that Ru active sites were well retained in the M‐MEA film and thus significantly contributed to its enhanced stability (Figure S15c and Table S7, Supporting Information). To evaluate the industrial application prospects of M‐MEA film, the HER performance at 50 °C was shown in Figure S16 (Supporting Information), with an overpotential of only 150 mV at 1 A cm^−2^, and can operate stably for more than 100 h under industrial‐grade current density (50 °C; 1 A cm^−2^), fully demonstrating the industrial application potential of this catalyst. Moreover, the peak of underpotential deposited H_2_ (H_UPD_) was clearly observable in the normalized electrochemical CV curves (Figure S17, Supporting Information). Compared with the H_UPD_ peak of the L‐MEA film and the H‐MEA film, respectively, that of the M‐MEA film was stronger. This result indicated that a larger amount of hydrogen atoms was adsorbed onto the M‐MEA film than onto the L‐MEA film and the H‐MEA film, respectively. Furthermore, compared with the position of the H_UPD_ stripping peak of the H‐MEA film and L‐MEA film, respectively, that of the M‐MEA film was markedly positively shifted (0.164 V (versus RHE) for the H‐MEA film, 0.169 V (versus RHE) for the L‐MEA film, and 0.151 V (versus RHE) for the M‐MEA film). This result demonstrated improved H desorption on the M‐MEA film, compared to the H‐MEA film and the L‐MEA film, respectively.

Structural and chemical stability are critical factors that significantly influence the lifetime of catalysts.^[^ ^74^ ^]^ In the current study, XRD, SEM, and AC‐HAADF‐STEM were carried out to examine the structure of M‐MEA film after stability testing. The XRD pattern (Figure S18a, Supporting Information) displayed distinct Ru diffraction peaks, which align with the findings observed prior to stability testing and thus demonstrate that the structure of M‐MEA film remained unchanged after stability testing. The SEM results (Figure S18b,c, Supporting Information) showed that all but a small amount of M‐MEA film remained attached to the carbon cloth after stability testing and maintained its original morphology. The EDS elemental mapping results (Figure S18d−d5, Supporting Information) revealed that the composition of Ru increased during the stability testing, possibly related to the leaching of Nb and Mn. The SAED pattern of the M‐MEA film after stability testing confirmed the presence of nanocrystalline Ru (Figure S18e−g, Supporting Information). Nevertheless, the size of the Mn‐rich phase increased during stability testing, with the average size increasing from 1.38 to 4.20 nm (Figure S18f, Supporting Information). This possibly occurred due to surface reconstruction, in keeping with Ostwald ripening theory. The transformation of sizes and compositions also induced small changes in the valence state and local atomic coordination, as reflected by XPS and XANES. The full XPS spectrum of the M‐MEA film after stability testing (Figure S7b, Supporting Information) showed that its surface continued to bear elemental Ru, Mn, and Nb, although the intensities of the signals for Mn and Nb were lower than they had been before stability testing. Similarly, the high‐resolution Ru 3p XPS spectrum for the M‐MEA film remained almost unchanged after stability testing (Figure S19a, Supporting Information). Meanwhile, the proportion of M–OH and adsorbed H_2_O on a catalyst indicates its affinity for H_2_O and aqueous media. Valence states were calculated for the XANES results, according to the fitted curves. The results show the valence state of Ru was not changed significantly by stability testing, i.e., it increased from 1.3 to 1.5 after stability testing (Figure 3e; Figure S9 and Table S2, Supporting Information). Besides, the EXAFS and the fitting results showed that from before to after stability testing, the coordination number of Ru–Ru/Nb decreased from 8.6 to 7.8, while the coordination numbers of Ru–O and Ru–Mn increased from 3.7 to 3.9 and 2.8 to 3.1, respectively (Figure 3h; Figure S19b and Table S3, Supporting Information). In addition, the varied coordination environment meant that from before to after stability testing, the Ru–O distance decreased from 2.055 to 2.032 Å. This result indicated close Ru–O coordination and coincided with the increased valence state of Ru. According to the above‐described characterization, the M‐MEA film presented excellent structural and chemical stability. Furthermore, it exhibited outstanding performance during long‐term and multiple‐cycle measurements, namely 1.2 mA cm^−2^ for 300 h and 25 000 cycles from 0 to 80 mA cm^−2^.

**Figure 5:**
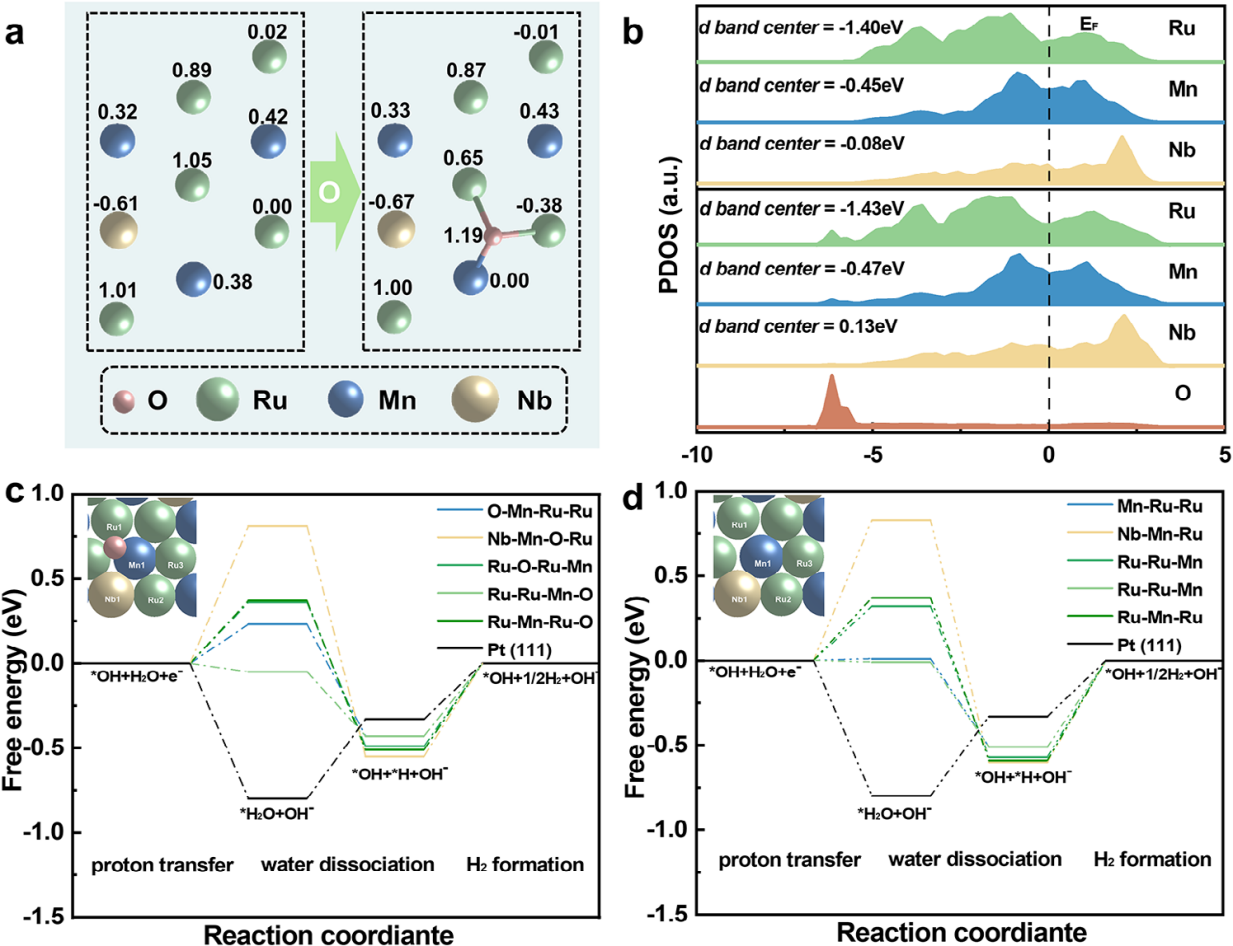
Density functional theoretical calculations. a) Bader charge analysis of RuMnNb and RuMnNbO models. b) Projected electronic density of states for d orbital of RuMnNb and RuMnNbO models. Step‐wise free energy profiles of HER at surfaces in RuMnNbO model c) and RuMnNb model d). The illustration in the figure shows the active sites of the corresponding model.

DFT calculations were used to explore the electronic distribution structures and corresponding kinetics mechanisms of the electrocatalysts. Two models were established, i.e., with and without embedding of O, to simulate the interfaces within M‐MEA film (Figure S20, Supporting Information). Bader charge analysis was conducted to determine electron interactions. As shown in **Figure** **5**a, Ru and Mn transferred more electrons to O with embedding of O than without embedding of O. In addition, with the embedding of O and the loss of electrons, d‐band center of Ru and Mn is proved by partial density of states to shift to lower energy and further away from to the Fermi energy level (Figure 5b). According to d‐band theory,^[^ ^75^ ^]^ a lower d‐band center induces higher occupancy of anti‐bonding states and more weakly adsorbs intermediates. Therefore, the adsorption of *H onto Ru and Mn was weakened, promoting the desorption process of HER. This is in accordance with the hydrogen underpotential deposition results, which showed a shift in the H_UPD_ peak from 0.169 V (versus RHE) for L‐MEA film to 0.151 V (versus RHE) for M‐MEA film (Figure S17d, Supporting Information). Conversely, the d‐band center of Nb was higher than the Fermi level, and the embedding of O caused it to shift to even higher energy, further reducing the occupation of anti‐bonding states and possibly enhancing adsorption of *H.^[^ ^76^ ^]^

The HER comprises the adsorption of H_2_O, H_2_O dissociation, as well as the adsorption and desorption of *H.^[^ ^77^ ^]^ Therefore, to clarify the energy changes of each reaction step in the HER, free energy diagrams were created. Previous studies and our experimental results (Figure S19a, Supporting Information) have revealed that activated hydroxide (*OH) is present at metal surfaces in the HER, even at negative potentials.^[^ ^78^, ^79^ ^]^ Thus, *OH sites could act as anchoring sites for proton transfer from the O‐rich interface to catalyst surfaces. Therefore, the adsorption energies of several *OH sites were calculated (Figure S21a,b and Table S10, Supporting Information). The results indicated that the adsorption energies of *OH on Ru sites adjacent to Nb were considerably greater than those on other Ru sites. Specifically, the adsorption energies of *OH on Ru2 and Ru3 were −1.14 and −0.62 eV, respectively, showing that Nb increased the *OH adsorption efficiency of Ru. In the first step of the HER, the transfer of proton to form H_2_O on Ru2 was exothermic, suggesting that proton transfer from liquid H_2_O to surfacial *OH was energetically favorable. In addition, although the presence of O increased the proton transfer energy barrier of Mn1 from 0.01 to 0.23 eV, the optimization of the Gibbs free energy (∆*G*H) decreased the total reaction energy barrier, which will be discussed in detail below. In the following reaction step, H_2_O dissociation on M‐MEA and Pt(111) differed significantly. On M‐MEA, H_2_O dissociation was exothermic, i.e., spontaneous, across all sites, regardless of the presence of interfacial O. However, on Pt(111), H_2_O dissociation was endothermic, i.e., non‐spontaneous (Figure 5c,d; Figure S21c and Table S11, Supporting Information). This difference demonstrated that compared with Pt(111), M‐MEA film was more effective at catalyzing H_2_O dissociation and thus generating a supply of *H. However, in contrast to the low‐energy‐barrier performance of Pt in the conversion of *H to H_2_, metallic phase of M‐MEA film performed poorly in the desorption of H_2_, leading to a disadvantageous ∆*G* for the entire H_2_ evolution process. The presence of interfacial O mitigated this problem. As shown in Figures S21d and S22 (Supporting Information), all O‐coordinated triple‐atom groups exhibited weaker H_2_ absorptivities than their corresponding purely metallic groups. This trend led to the activation of multiple routes, such as the formation of –O–Mn–Ru–Ru– and –Ru–Ru–Mn–O– species, and reduced these routes’ energy barriers throughout the HER. These results are also consistent with the differences observed between the behaviour of M‐MEA film in acidic solution and its behavior in alkaline solution. For example, in acidic solution, the oxide‐based structure of M‐MEA film tended to dissolve, and metallic M‐MEA film remained, resulting in an increase in the energy barrier for H_2_ evolution. In summary, the above‐described simulation results accounted for the intrinsically excellent properties of the M‐MEA and explained why the regulation of interfacial O led to its showing excellent H_2_ evolution performance.

## 3 Conclusion

In this study, we presented a one‐step design and fabrication approach to create a Ru‐based catalyst, RuMnNbO, with a crystal–crystal nano‐dual‐phase structure. This structure was characterized by the presence of an Mn‐rich phase (≈1.3 nm) embedded in Mn‐poor crystals, together with an O‐rich interface. Thus, RuMnNbO was a supra‐nano solid‐solution film and it was found to demonstrate remarkable HER performance and exceptional sustainability. Its high HER performance may be attributed to synergistic effects of the Mn‐rich/poor dual phase, which facilitated the formation of the O‐rich interface and ensured that there was an ultrahigh density of active interfaces. Moreover, the M‐MEA film form of RuMnNbO exhibited exceptional stability, as illustrated by its operating continuously for at least 300 h under alkaline conditions at 1.2 A cm^−2^.

Overall, this study developed a high‐efficiency and robust HER catalyst for enhanced industrial H_2_ production. Meanwhile, it also introduced a novel approach for achieving a delicate balance for catalytic stability and activity. These findings pave a path for the design of future catalysts aimed at sustainable energy development.

## 4 Experimental Section

### Materials

Raw element of Ru (99.95 wt.%), Mn (99.9 wt.%), Nb (99.95 wt.%) were provided by Sante Material Technology Co., Ltd (China Taizhou). Commercial Pt/C (20 wt.%) supplied by Macklin (Shanghai, China). Analytical grade sulfuric acid (H_2_SO_4_) and potassium hydroxide (KOH) were provided by Aladdin (Shanghai, China). Absolute ethanol and copper sulphate (CuSO_4_) were supplied by Shanghai Sinopharm Chemical Reagent Ltd. Co of China.

### Fabrication of the M‐MEA Film

The M‐MEA catalyst was synthesized by one step magnetron co‐sputtering, with Ru (99.95%), Mn (99.9%), Nb (99.95%) as the sputtering targets. A crystal–crystal nano‐dual‐phase film (Ru62Mn12Nb21O5 (at%) thickness ≈750 nm) was deposited on single grain oriented Si (001) and clean carbon cloth by magnetron co‐sputtering. The background vacuum was 5 × 10^−5^ Pa. During the co‐sputtering, the argon pressure kept 0.4 Pa, and the deposition rate remained at 12.5 nm min^−1^, the bias voltage kept −60 V, and the substrate temperature kept at ambient temperature. The comparative samples were also prepared under the similar process.

### Structural and Compositional Characterization

X‐ray diffraction (DMAX‐2500 PC) were carried out to identify the crystal phase of the M‐MEA film. The film surface morphology of M‐MEA was analyzed by a scanning electron microscope (FESEM, SU‐70, Hitachi, Japan and FE‐SEM, Gemini 500, ZEISS). The microstructure was further investigated by high‐resolution transmission electron microscope (HRTEM, JEOL JEM F200) along with selected area electron diffraction (SAED) and energy dispersive spectrometry (EDS). High‐resolution (Atomic scale) scanning transmission electron microscopy (STEM) images and corresponding EDS mappings were acquired by AC‐TEM, FEI‐Themis Z. 3D atomic probe tomography (APT) characterization was performed using by LEAP 5000HR. The AC‐TEM and APT specimens were fabricated by FEI focused ion beam/scanning electron microscope (FIB/SEM, Thermofisher Scios 2). X‐ray photoelectron spectroscopy (XPS) was performed by Thermo Scientific K‐Alpha instrument to determine valence state. Inductively coupled plasma‐optical emission spectrometry (ICP‐OES) was conducted to determine composition of fresh film as well as leached metallic ions during stability experiments (Thermo Fisher iCAP PRO).

The X‐ray absorption find structure spectra (Ru K‐edge) were gathered at the BL 14W1 beamline of the Shanghai Synchrotron Radiation Facility, the data collection was carried out in fluorescence excitation mode using ionization chamber for Ru foil using a Lytle detector for two samples. All spectra were gathered in ambient conditions. The XAFS data were processed according to the standard procedures using the Athena module implemented in the IFEFFIT software packages. The EXAFS spectra were acquired by subtracting the post‐edge background from the overall absorption and then normalizing with respect to the edge‐jump step. Subsequently, the *χ(k)* data of were Fourier transformed to real (R) space using a hanning windows (*dk* = 1.0 Å^−1^) to separate the EXAFS contributions from different coordination shells. To acquire the quantitative structural parameters around central atoms, least‐squares curve parameter fitting was carried out using the ARTEMIS module of IFEFFIT software packages.^[^ ^80^, ^81^ ^]^ For Wavelet Transform analysis, the *χ(k)* exported from Athena was imported into the Hama Fortran code.^[^ ^82^ ^]^ The following was the list of parameters: *R‐*range, 0.0 – 4.0 Å, *k‐*range, 0 – 16.0 Å^−1^ for sample and Standards; *k* weight, 2; and Morlet function with *κ* = 8, *σ* = 1 was used as the mother wavelet to provide the overall distribution.

### Electrochemical Measurements

Throughout the measurement process, the electrochemical catalytic performance was obtained in 1 M KOH solution by an electrochemical workstation (CHI 760E) comprising standard three electrodes. The workstation includes the working electrode, the countering electrode (graphite rod, d  =  6 mm), and the reference electrode (saturated calomel electrode (SCE)). The potential mentioned in this work was calibrated using reversible hydrogen electrode based on the Nernst equation with a correction of *iR* (*i* is current, *R* is the resistance of solution) loss. To further ensure the accuracy of value, the SEC was calibrated in H_2_‐saturated 1 M KOH solution (Figure S18, Supporting Information) by scanning rate of 5 mV s^−1^. Normalize all polarization curves according to their geometric surface area. For comparison, Pt/C (20 wt.%) catalysts and Pt sheet (1×1 cm) were tested. The Pt/C catalyst inks were carefully loaded on carbon cloth (1 cm^2^), the mass load of Pt is 0.6 mg cm^−2^.

Electrochemical impedance spectroscopy (EIS) was employed (10^5^‐0.1 Hz). Double layer capacitance (*C*_dl_) was roughly identified by cyclic voltammetry with different scanning rates from 20–120 mV s^−1^ in the non‐faradaic potential region. The electrochemical surface areas (ECSA) were obtained base ECSA = *C*_dl_/*C*_s_, where *C*_s_ represents the specific capacitance and *C*_dl_ represents measured double‐layer capacitance. In this study, *C*_s_ = 0.04 mF cm^−2^. The HER stability was tested under 100 and 1200 mA cm^−2^. The turnover frequency of M‐MEA film and Pt/C was evaluated by the equation:

(1) $$TOF=\frac{i}{2Fn}=\frac{\left|j\right|A}{2Fn}$$

where *i* represents the current (mA) from polarization curve (LSV) in Figure 4a,n (mmol) represents the mole value of active sites at the surface, *F* represents the Faraday constant (96 485 C mol^−1^) and *A* represents the geometric area (cm^2^), **|** *j* **|** is the absolute value of current density (mA cm^−2^), and the coefficient 1/2 is because one hydrogen molecule needs two electrons.

The molar value of active sites was reflected through the deposition of Cu^2+^/Cu. As shown in Figure S24 (Supporting Information), the anodic peak around 0.38 V (versus RHE) was attributed to the Cu stripping peaks. The deposition of copper changes the area of the cycling voltammetry. Based on this difference, the quantity of active sites can be roughly estimated following the following formula:

(2) $$n=\frac{Q_{Cu}}{2F}$$

where *Q_Cu_* is the difference in the area of cycling voltammetry. The value of active sites of M‐MEA film was 1.66 mC, and the value for commercial Pt/C catalyst was 8.76 mC. Therefore, the TOF value was

For M‐MEA film,

(3) $$TOF=\frac{i}{2Fn}=\frac{\left|j\right|*1}{1.66\;mC}=0.6\left|j\right|$$

For Pt/C,

(4) $$TOF=\frac{i}{2Fn}=\frac{\left|j\right|*1}{8.76\;mC}=0.11\left|j\right|$$

### Density Functional Theory Calculation

DFT calculations analysis were employed by the Vienna Ab‐initio Simulation Package (VASP). Plane waves were generated using projector augmented‐wave (PAW) potentials. The exchange‐correlation effect was illustrated by the Perdew‐Burke‐Ernzerhof (PBE) functional. The Grimmes D3 method was utilized for the dispersion energy between absorbates and surfaces. The Methfessel‐Paxton method was carried out for electron smearing with a width of 0.2 eV. The self‐consistent field loop was set to converge at an energy below 1×10^−6^ eV, and the maximum force of the unconstrained atoms was less than 0.02 eV Å^−1^ for geometry optimization processes.

The Ru_21_Mn_9_Nb_6_ were modeled with the Ru primitive cell, and k‐point and cut‐off energy were set as 12×12×6, 400 eV for the cell optimization. Based on X‐ray diffraction (XRD) results, the Ru_21_Mn_9_Nb_6_(001) surface was chosen for evaluating the catalytic ability, including three elemental steps: water dissociation, OH* desorption, and H_2_ formation. Mn and Nb were randomly substituted for Ru in the Ru(001) structure to form Ru_21_Mn_9_Nb_6_. The surfaces were modeled using 3×3 supercell and vacuum layer of 15 Å. A k‐point of 3×3×1 was used for all surface calculations. For comparison, the Pt(111) surface was also modeled (2×2 supercell with 4 atomic layers), and calculated with 4×4×1 k‐point. Note that the bottom two layers of Ru_21_Mn_9_Nb_6_(001) and Pt(111) were fixed in all geometry optimization processes. The stepwise free energy profile of the alkaline HER was built by following the computational hydrogen electrode (CHE) method proposed by Norskov and co‐workers. The Gibbs reaction free energy (Δ*G*) was obtained with Formula (5):

(5) $$\Delta G=\Delta E_{\mathrm{DFT}}+\Delta ZPE+T\Delta S+\Delta E_{\mathrm{sol}}$$

where Δ*E*_DFT_ is the DFT calculated reaction energy, Δ*ZPE* and *T*Δ*S* is the energy correction for zero‐point energy and entropy, Δ*E*_sol_ is the solvation energy correction, which was found to be ‐0.32 eV and ‐0.38 eV for bridge, hollow site OH*, ‐0.36 eV for site H_2_O*, and ‐0.07 eV and ‐0.12 eV for top, hollow site H*, according to work by Goddard and co‐workers.

## Conflict of Interest

The authors declare no conflict of interest.

## Author Contributions

Conceptualization: J.Lu., S.D.Liu. Research plan: J.Li., H.K.Li., S.D.Liu., and J.Liu. Prepared samples: J.Li. and J.Yu. Electrochemical experiments: J.Li., L.Ren. X‐ray photoelectron spectroscopy analyses: H.K.Li. and Y.Y.Yang. HAADF‐STEM analyses: J.Li. and X.F.Liu. 3D atom probe tomography: J.Li., X.F.Liu., and Z.Jia. Density functional theory simulations: X.Wang., K.Xu. and K.K.Chang. Writing—original draft: J.Li., H.K.Li., and S.D.Liu. Writing—review and editing: All of the authors.

## Supporting information



Supporting Information

## Data Availability

The data that support the findings of this study are available from the corresponding author upon reasonable request.
