# Supplementary material for: A Nanostructured Ru‐Mn‐Nb Alloy with Oxygen‐Enriched Boundaries for Ampere‐Level Hydrogen Evolution
Source: Adv Sci (Weinh). 2025 Apr 26;12(27):2501976. doi: 10.1002/advs.202501976 (PMC12279174; doi:10.1002/advs.202501976)
Supplement: Supplementary file 1 — Supporting Information [file ADVS-12-2501976-s001.docx]

**A Nanostructured Ru-Mn-Nb Alloy with Oxygen-Enriched Boundaries for Ampere-Level Hydrogen Evolution**

Jie Li^1^, Xue Wang^2^, Jun Yu^3^, Kai Xu^2^, Zhe Jia^4^, Hongkun Li^5,^*, Lei Ren^1^, Yiyuan Yang^4^, Keke Chang^2,^*, Yangyang Li^5,^*, Xiangfa Liu^1,^*, Jian Lu^5,^*, Sida Liu^3,^*

1. *Key Laboratory for Liquid-Solid Structural Evolution & Processing of Materials, Ministry of Education, Shandong University, Jinan, 250061, China.*
2. *Key Laboratory of Advanced Marine Materials, Ningbo Institute of Materials Technology and Engineering, Chinese Academy of Sciences, Ningbo, Zhejiang 315201, China.*
3. *Laboratory for Multiscale Mechanics and Medical Science, SV LAB, School of Aerospace, Xi'an Jiaotong University, Xi'an 710049, China.*
4. *School of Materials Science and Engineering, Jiangsu Key Laboratory for Advanced Metallic Materials, Southeast University, Nanjing, 211189, China.*
5. *Hong Kong Branch of National Precious Metals Material Engineering Research Center, City University of Hong Kong, Hong Kong SAR, China.*

* Corresponding authors: [li.hongkun@cityu.edu.hk](mailto:li.hongkun@cityu.edu.hk), [changkeke@nimte.ac.cn,](mailto:changkeke@nimte.ac.cn,) [yangli@cityu.edu.hk,](mailto:yangli@cityu.edu.hk,) [xfliu@sdu.edu.cn,](mailto:xfliu@sdu.edu.cn,) [jianlu@cityu.edu.hk](mailto:jianlu@cityu.edu.hk), sidaliu@xjtu.edu.cn


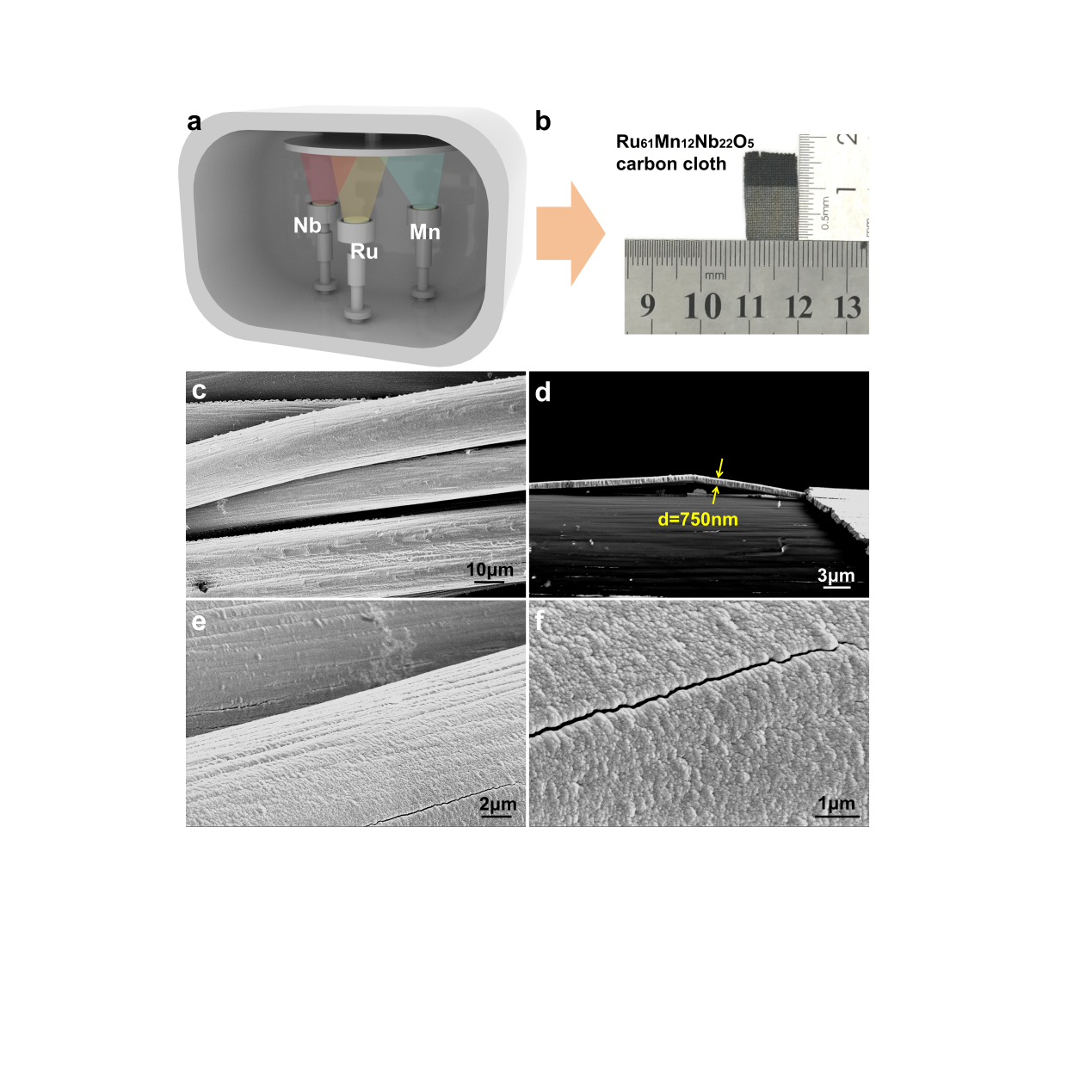


**Supplementary Fig. 1.** Materials synthesis diagram (a) and photographs of the M-MEA film (b). (c–f) SEM images of the as-dep M-MEA film deposited on carbon cloth under various magnifications.

**
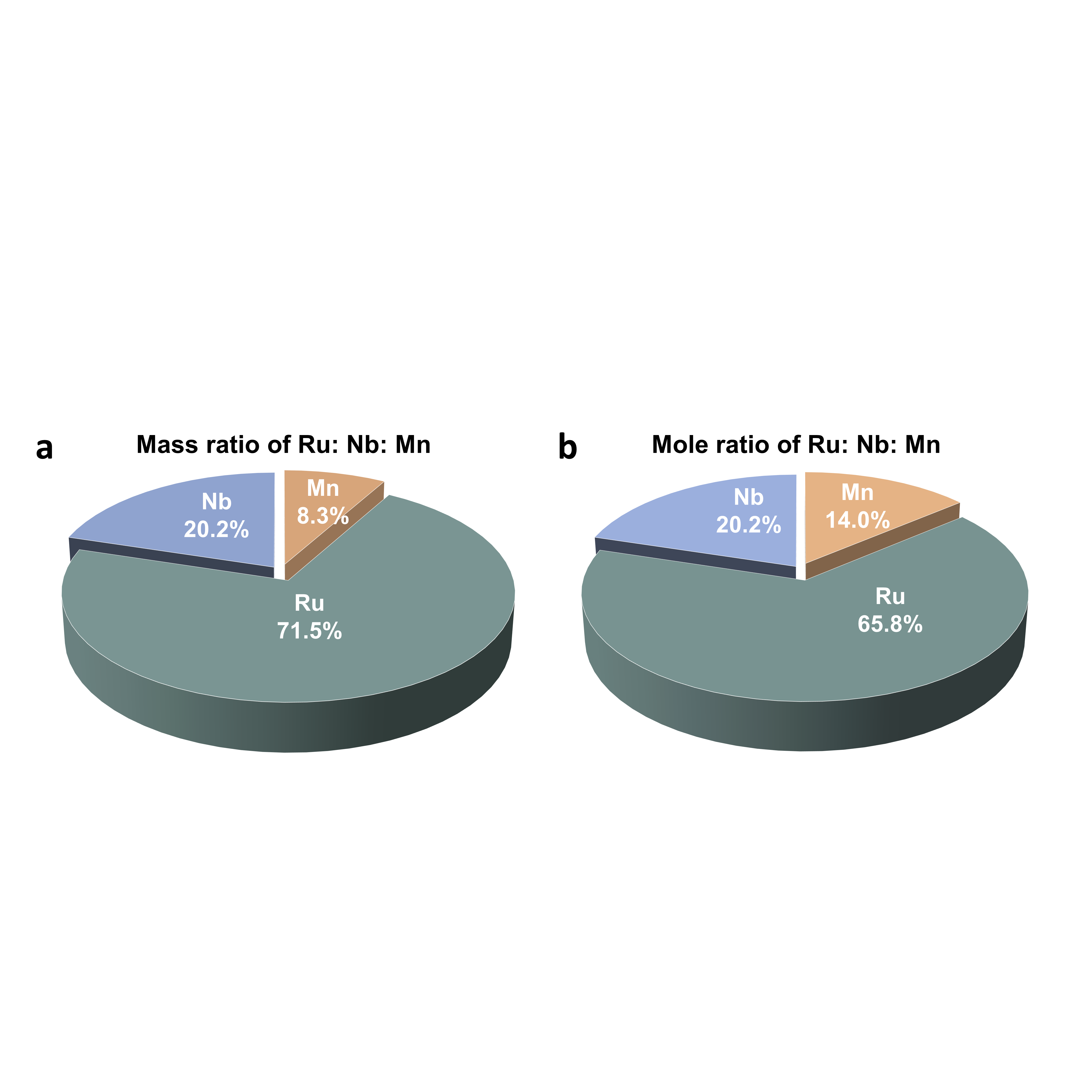
**

**Supplementary Fig. 2.** Pie charts of mass (a) and mole (b) ratio obtained from ICP analysis for M-MEA film.

**
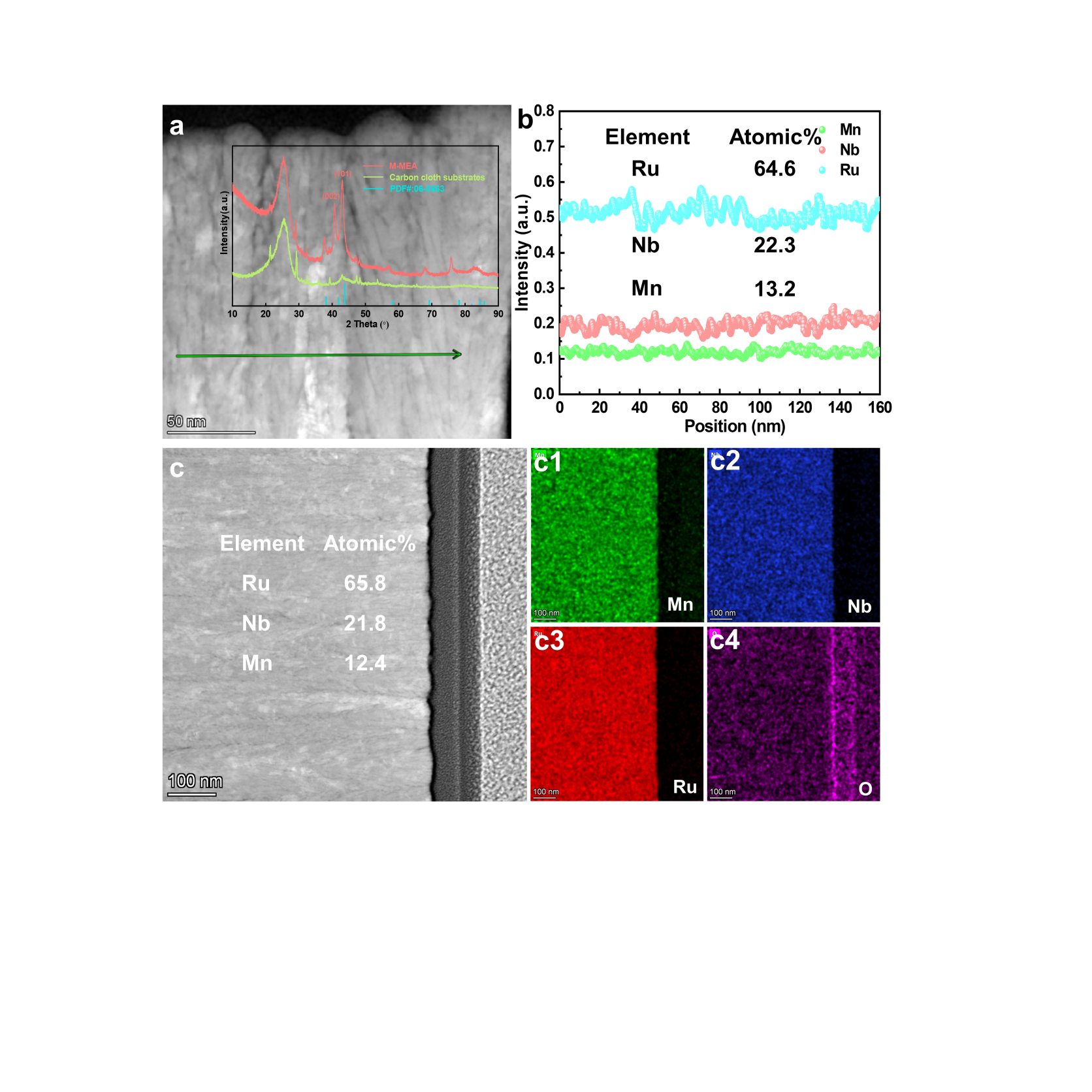
**

**Supplementary Fig. 3.** Elemental investigation. (a, b) EDS line scan analysis and EDS mapping (c–c4) of the as-dep M-MEA film under TEM.

**
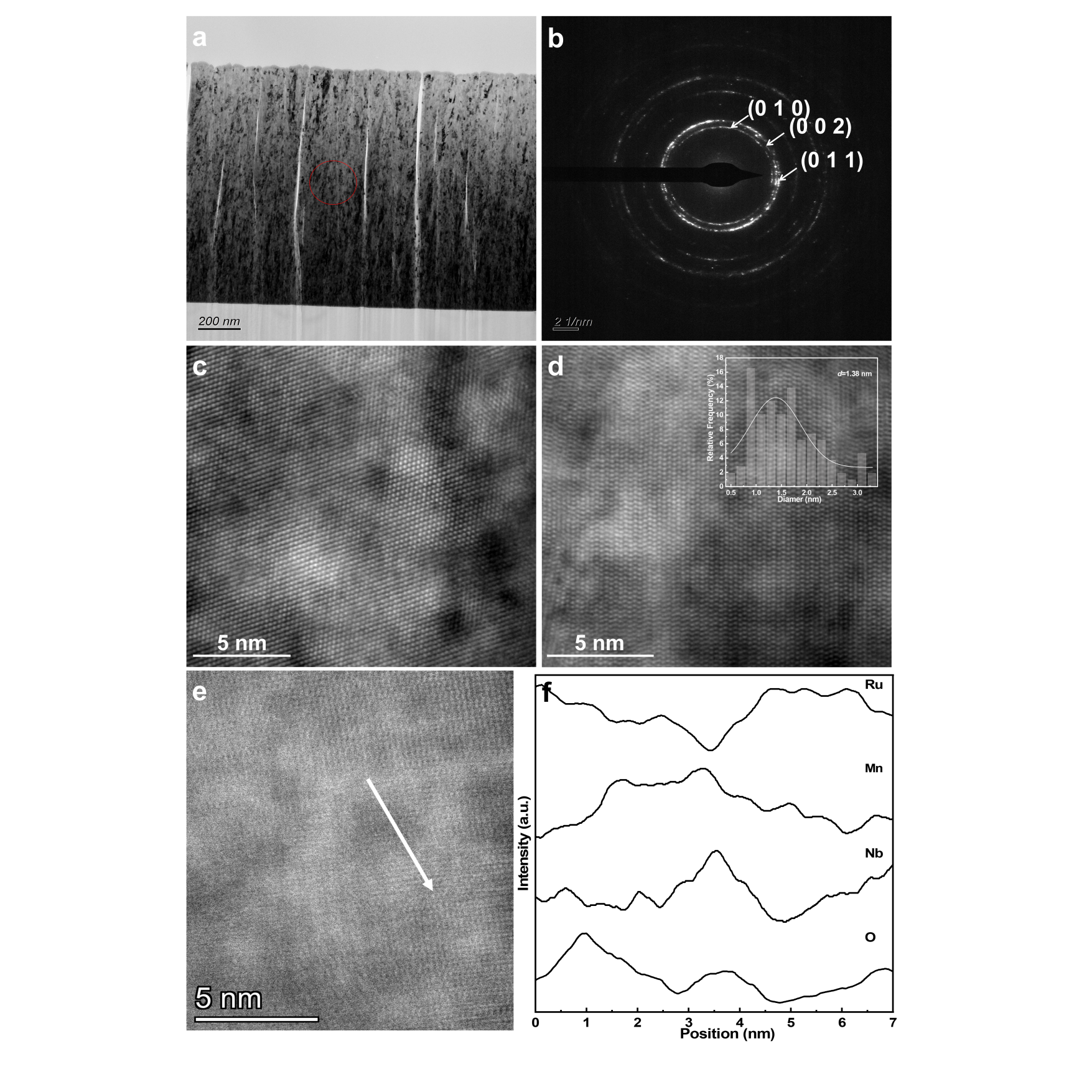
**

**Supplementary Fig. 4.** Selected area electron diffraction (SAED) pattern of the M-MEA film (a, b). TEM images of the as-dep M-MEA film under various magnifications and statistical chart of Mn rich phase size (c, d). EDS line scan analysis of the as-dep M-MEA film under high magnification TEM (e, f).

**
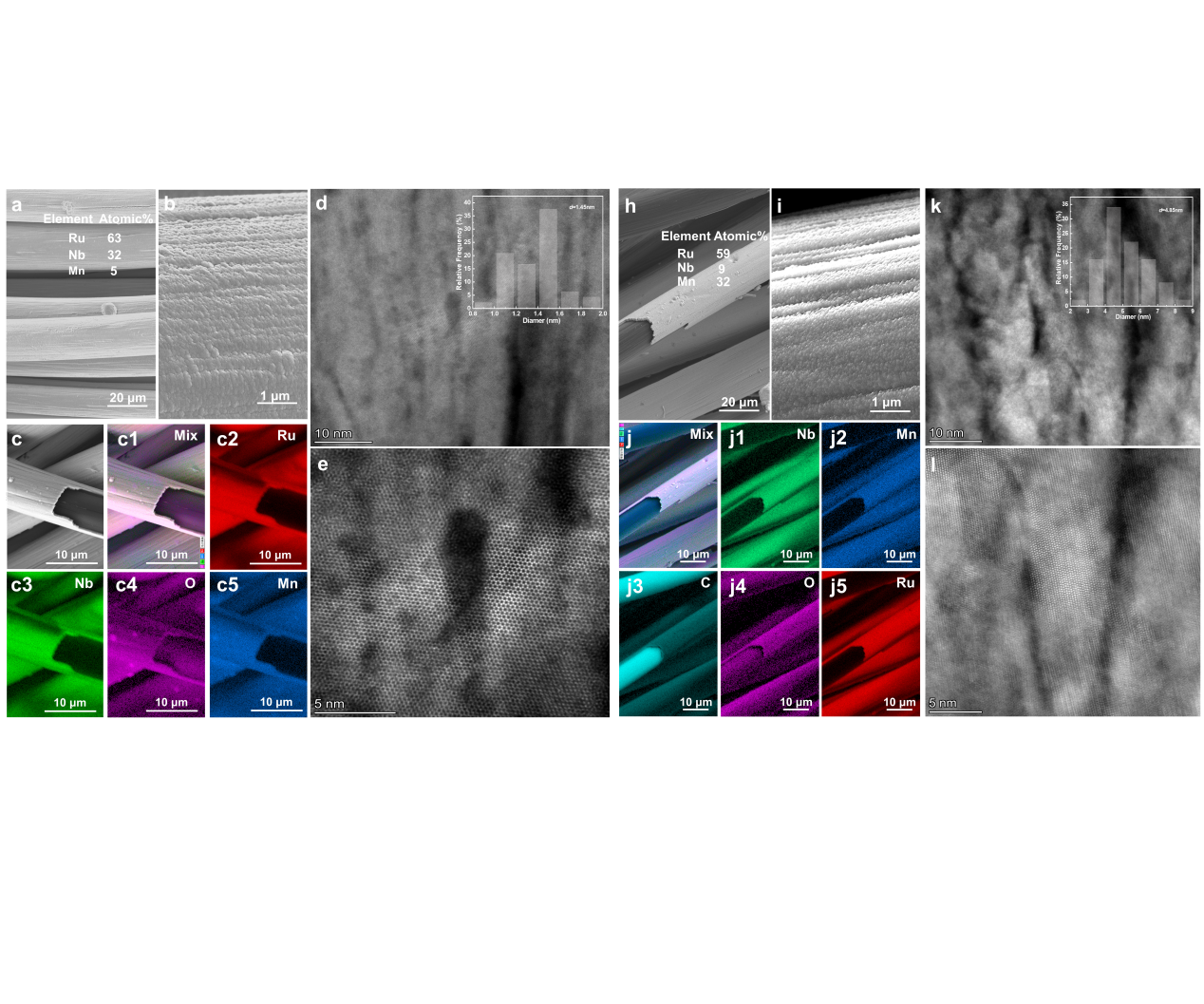
**

**Supplementary Fig. 5.** H-MEA film (Ru: Mn: Nb = 59: 32: 9) characterization. SEM images (a, b) of the as-dep H-MEA film under various magnifications. EDS mapping (c–c5). TEM images and statistical chart of Mn rich phase size (d, e). L-MEA film (Ru: Mn: Nb is 63: 5: 32) characterization. SEM images (h, i) of the as-dep L-MEA film under various magnifications. EDS mapping (j–j5). TEM images and statistical chart of Mn rich phase size (k, l).

**
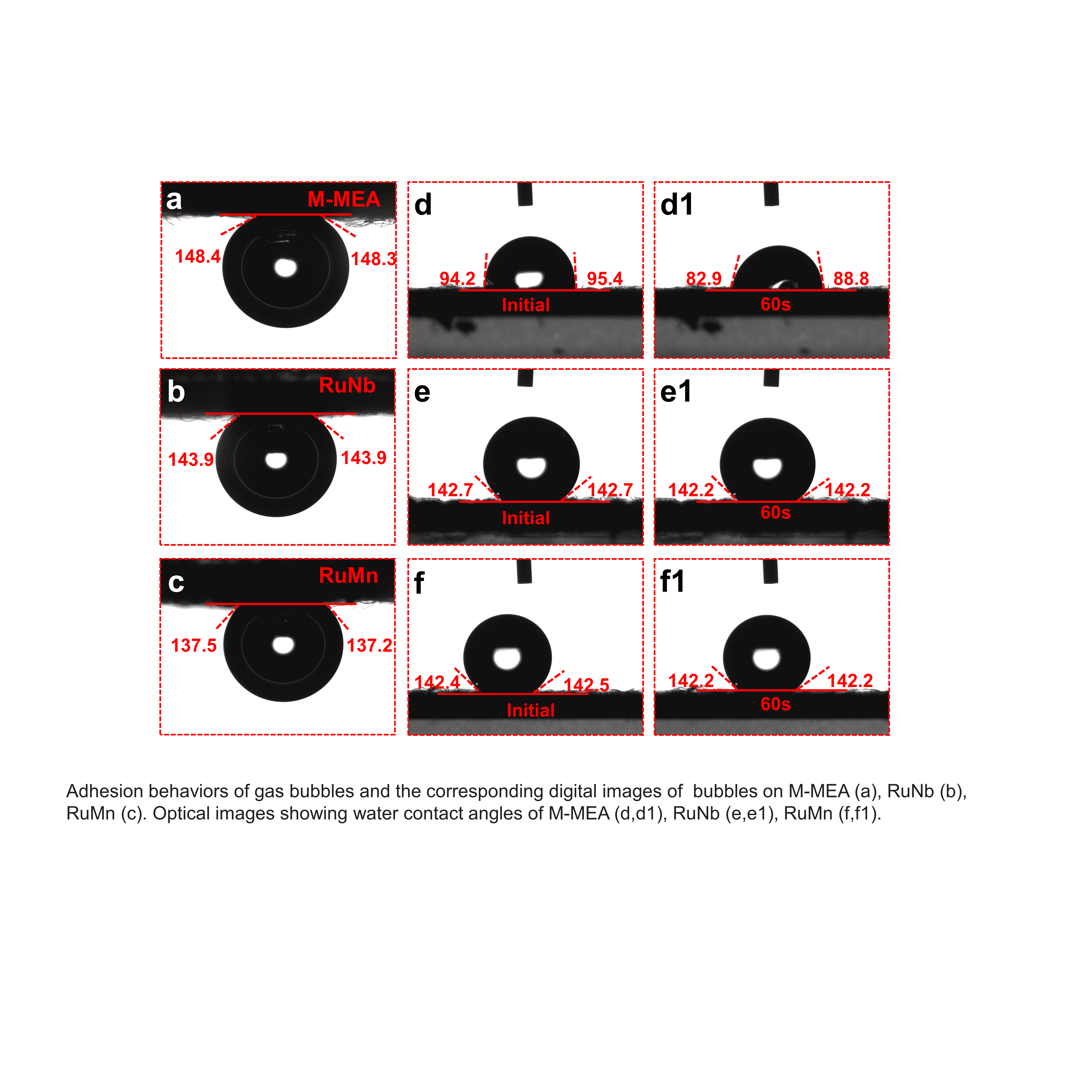
**

**Supplementary Fig. 6.** Adhesion behaviors of gas bubbles and the corresponding digital images of bubbles on M-MEA (a), RuNb (b), RuMn (c). Optical images showing water contact angles of M-MEA (d, d1), RuNb (e, e1), RuMn (f, f1).

**
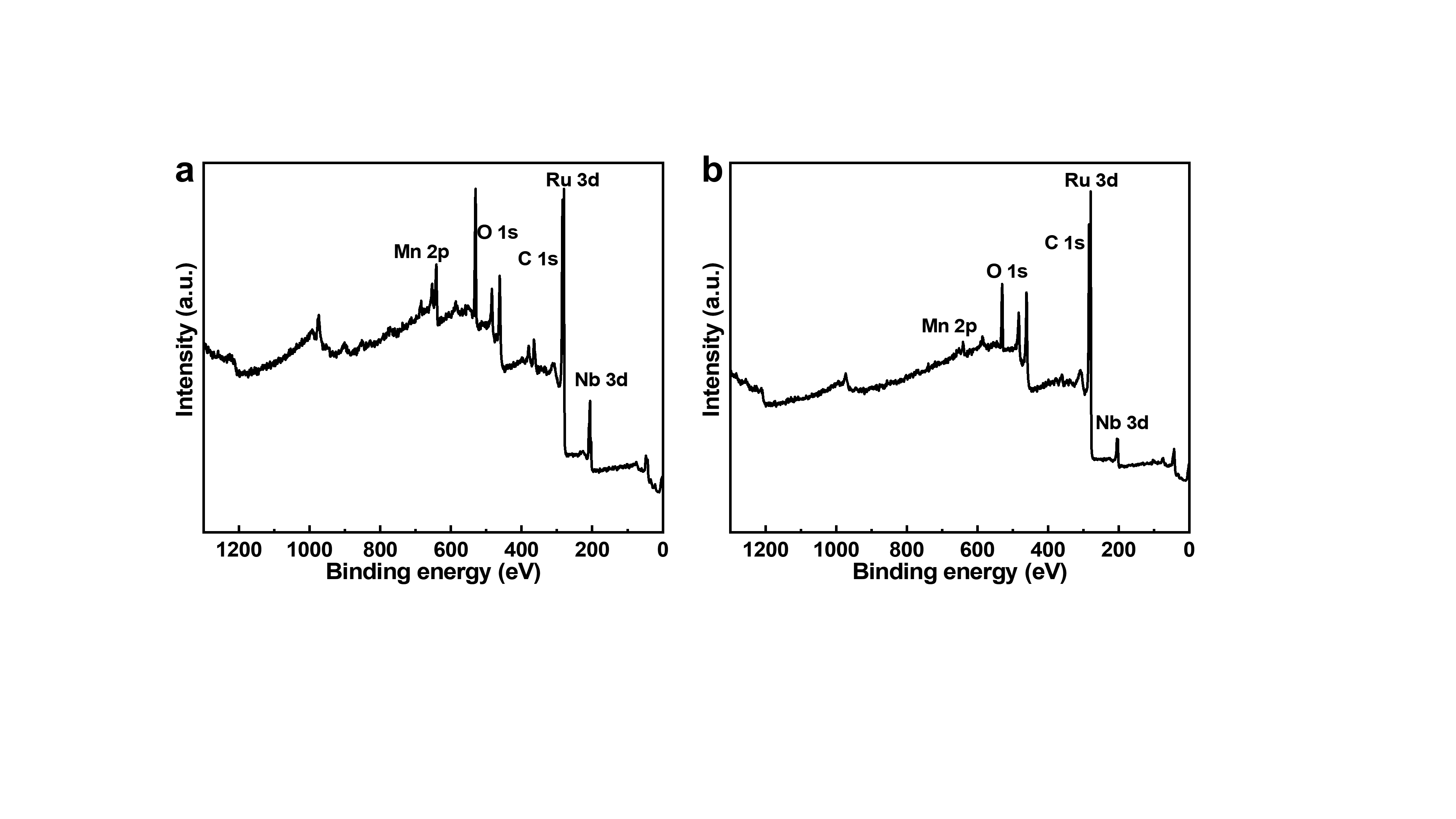
**

**Supplementary Fig. 7.** XPS survey scan of fresh M-MEA film (a) and after stability (b).

**
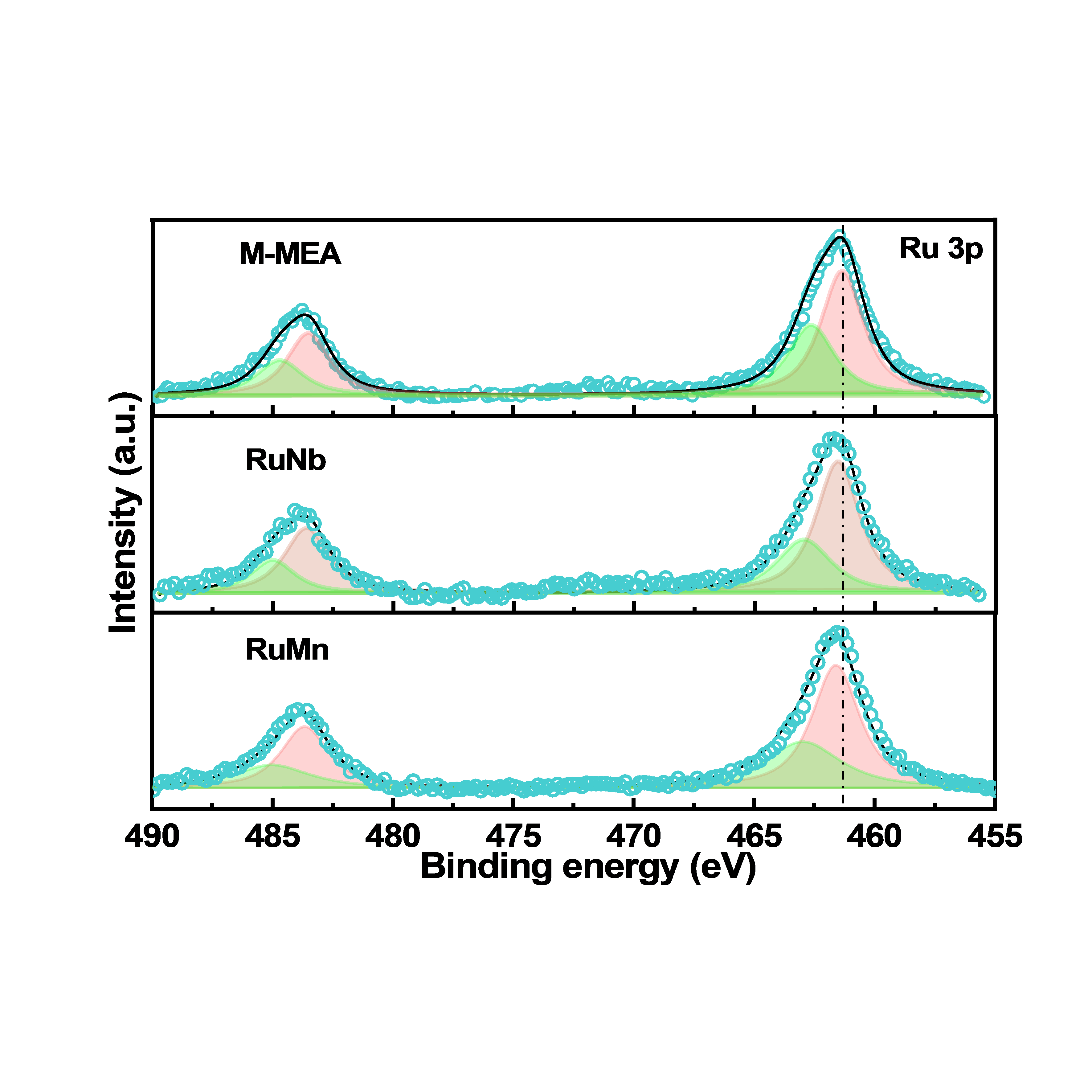
**

**Supplementary Fig. 8.** XPS spectra of Ru 3p for M-MEA film and binary films.

**
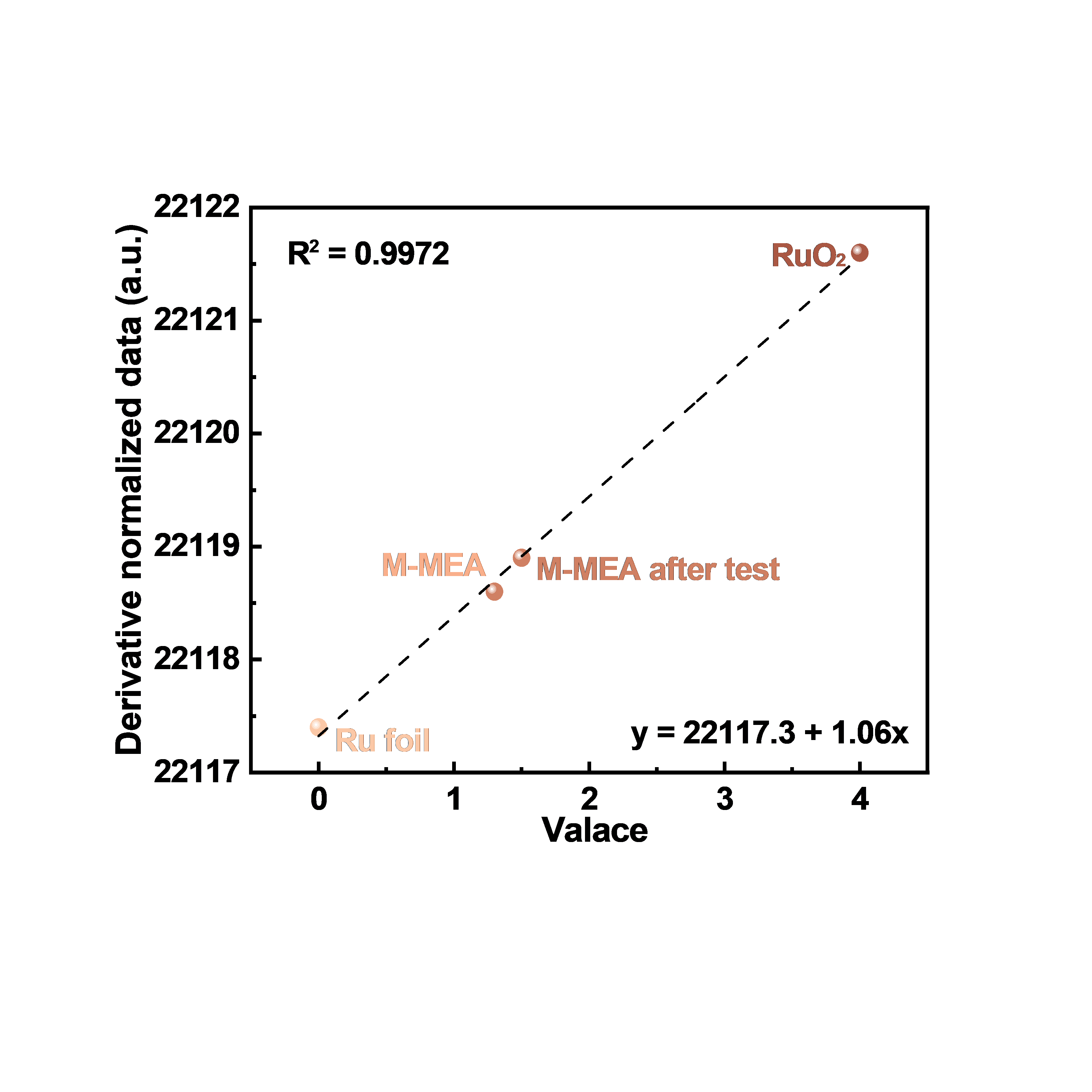
**

**Supplementary Fig. 9.** Ru k-edge energies for Ru foil, RuO2, fresh M-MEA and after stability films as a function of the Ru valence.

**
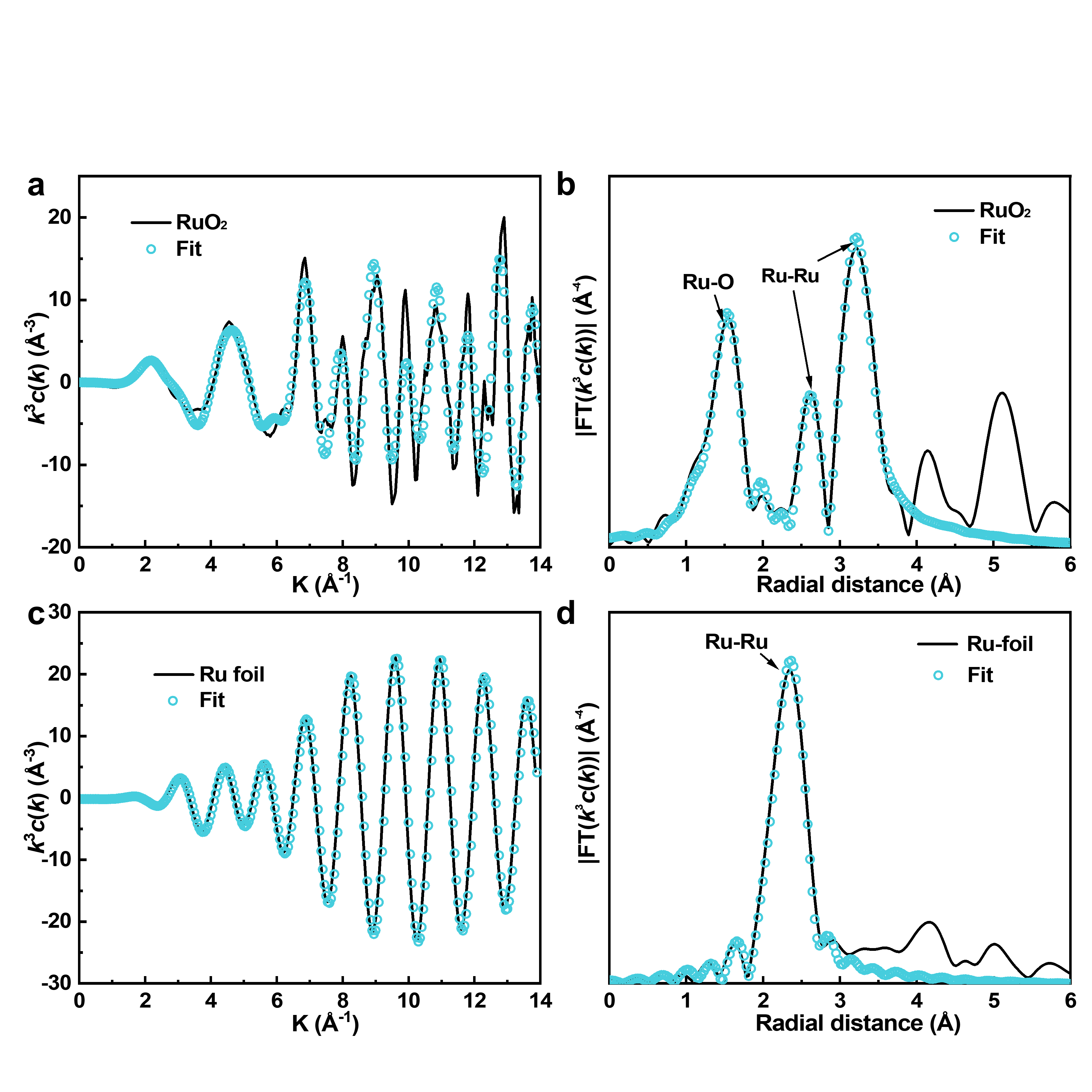
**

**Supplementary Fig. 10.** EXAFS k-space and R-space fitting curves of RuO2 foil K-edge (a, b) and Ru (c, d).

***

***

**Supplementary Fig. 11.** (a) The potential at 0 mA is 1.072 V. Therefore, the equation for transferring SCE to RHE potential was confirmed to be E_(_*_RHE_*_)_=E_(_*_SEC_*_)_+1.072V. Electrocatalytic performance of the H-MEA, M-MEA and L-MEA samples in 1M KOH solution. (b) Linear sweep voltammetry at a scanning rate of 5 mV/s (with *iR* loss correction). (c) Nyquist plots of the catalysts. (d) The Tafel slopes of the three catalysts. (e–g) The cyclic voltammetry of the three samples at different scanning rates (20 to 120 mV/s with 20 mV/s intervals) among 0.05–0.15 V (*vs.* RHE). (h) Linear fitting of the capacitive currents versus cyclic voltammetry scans for three catalysts.

**
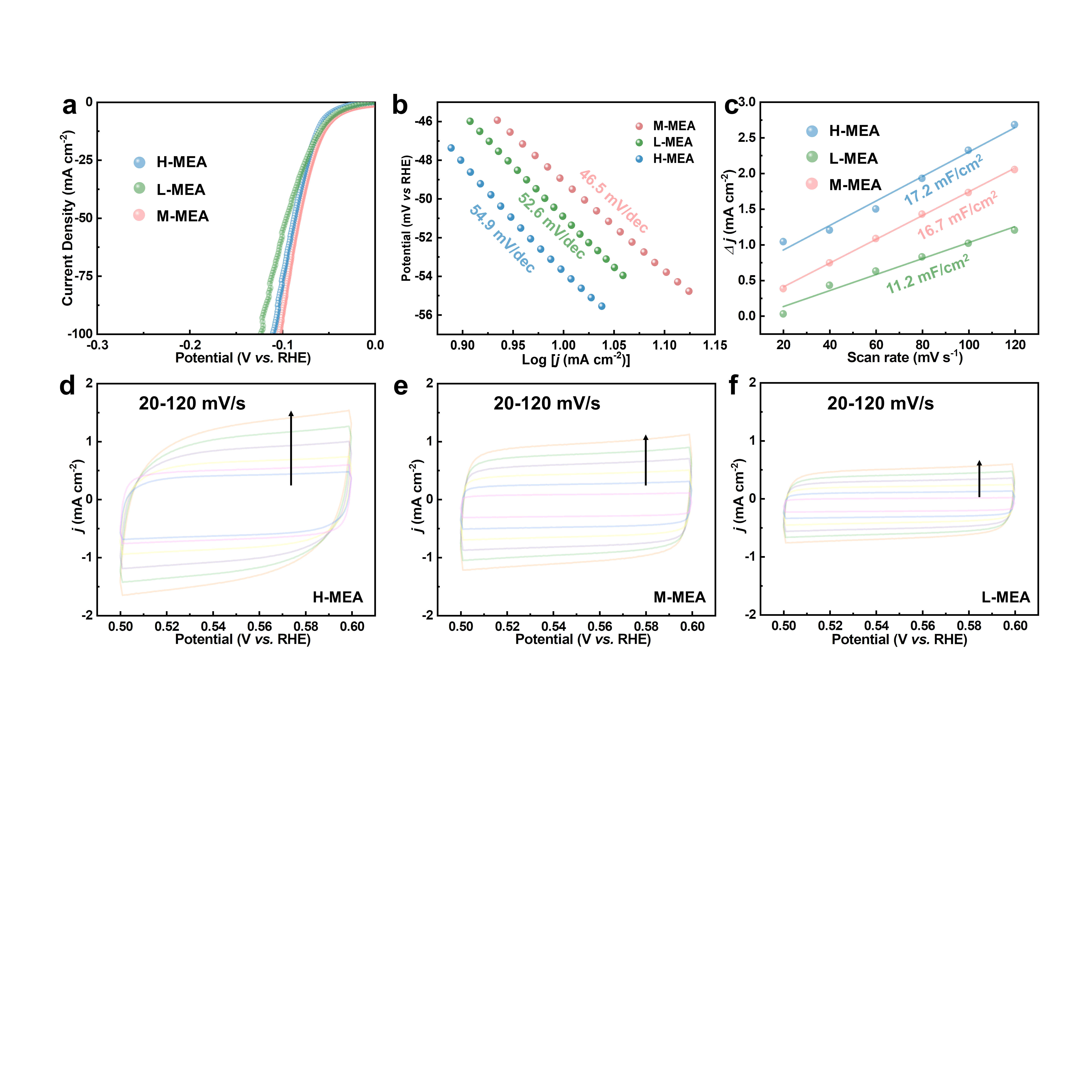
**

**Supplementary Fig. 12.** Electrocatalytic performance of the H-MEA, M-MEA and L-MEA samples in 0.5M H2SO4 solution. (a) Linear sweep voltammetry at a scanning rate of 5 mV/s (with *iR* loss correction). (b) The Tafel slopes of the three catalysts tested in 0.5M H2SO4. (c) Linear fitting of the capacitive currents versus cyclic voltammetry scans. (d–f) The cyclic voltammetry of the three samples at different scanning rates (20 to 120 mV/s with 20 mV/s intervals) among 0.5–0.6 V (*vs.* RHE).


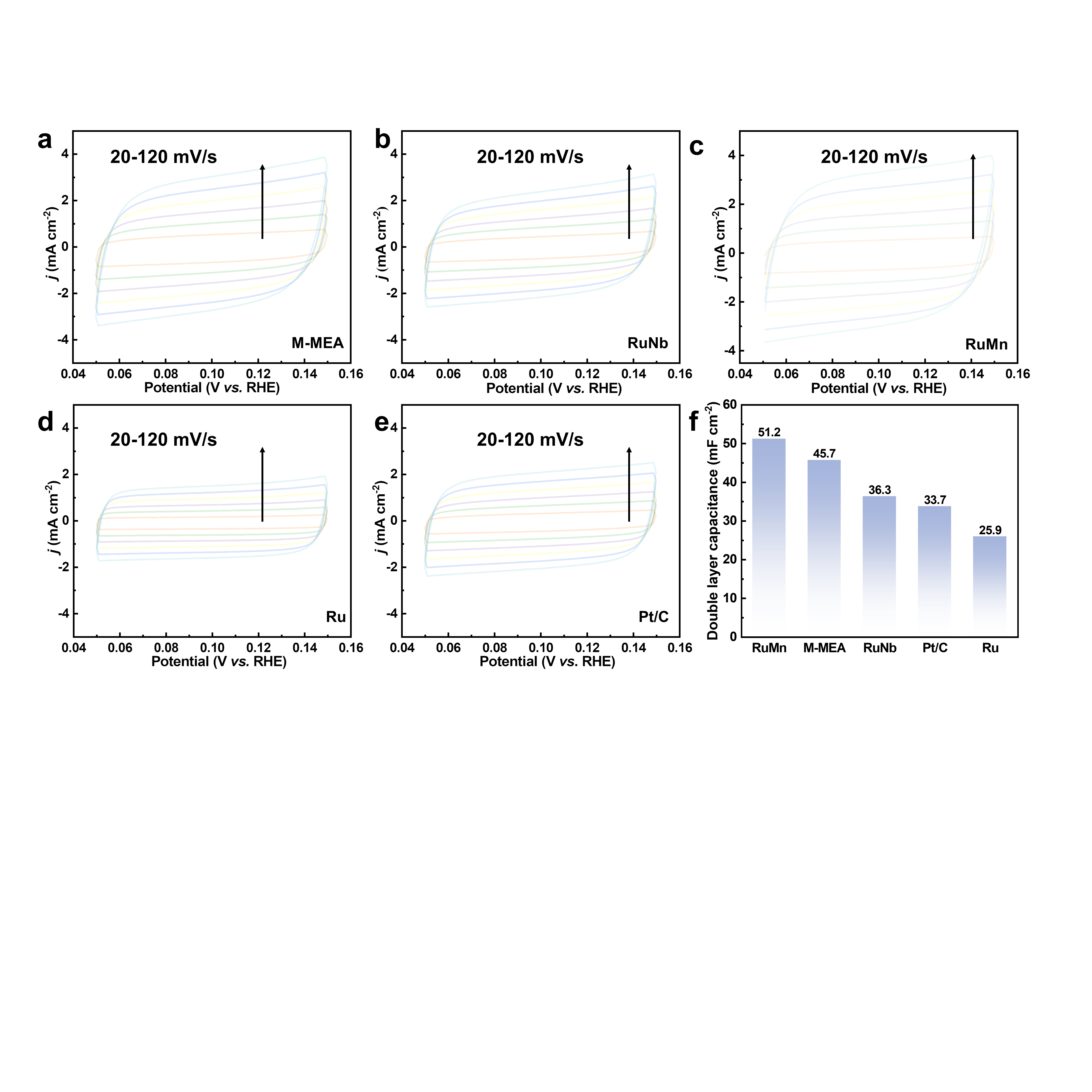


**Supplementary Fig. 13.** ECSA measurements of electrocatalysts. Electrochemical cyclic voltammetry scans recorded for (a) M-MEA. (b) RuNb. (c) RuMn. (d) Ru and (e) Pt/C. Scan rates are 20, 40, 60, 80, 100 and 120 mV/s among 0.5–0.6 V (*vs.* RHE). (f). Statistics of double-layer capacitors.


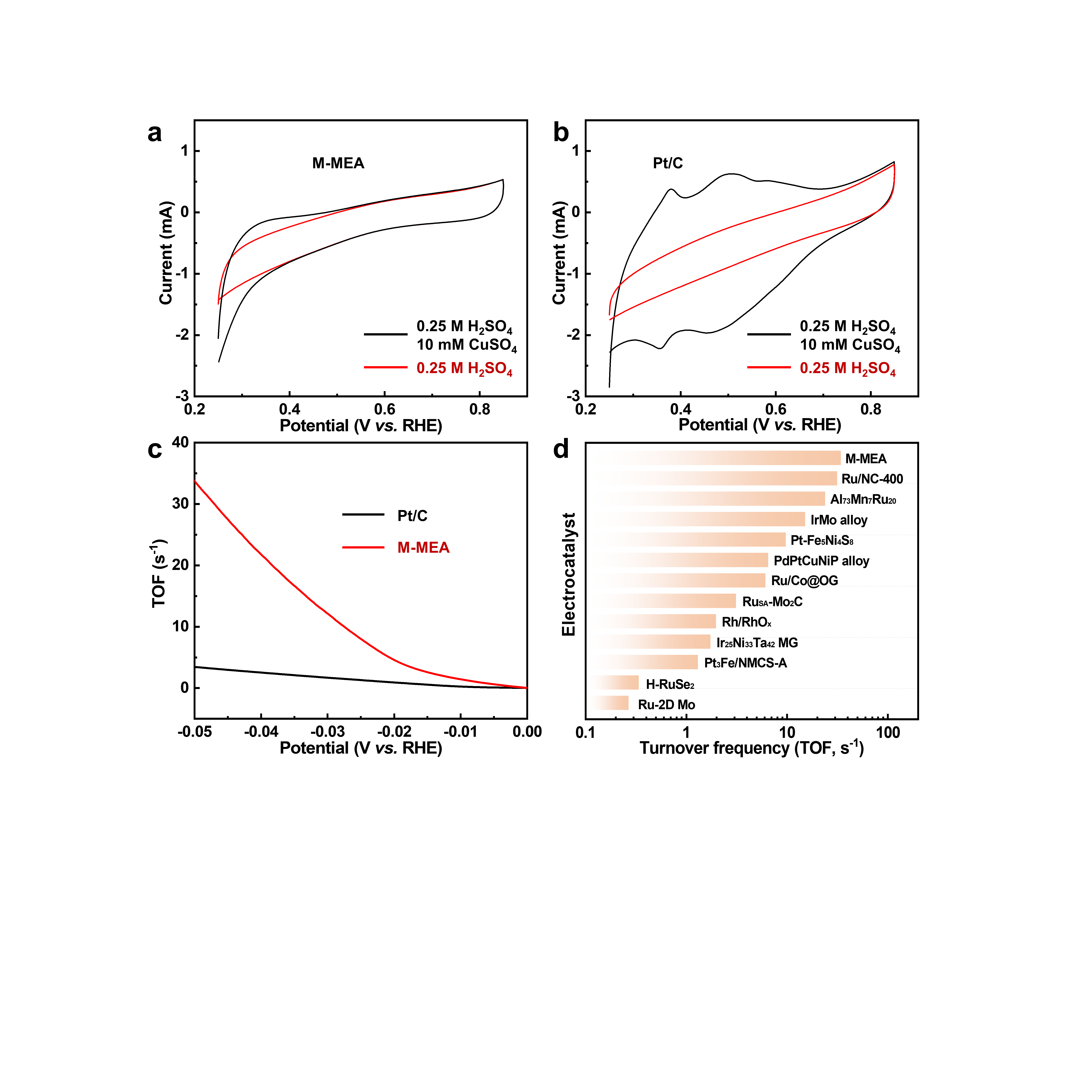


**Supplementary Fig. 14.** Turnover frequencies measured by cycling voltammetry of different solutions at 10 mV/s. (a) M-MEA film. (b) Commercial 20 wt.% Pt/C. (c) TOF curve based on current density in Fig. 4a. (d) TOF value comparison among different noble alloy catalyst.


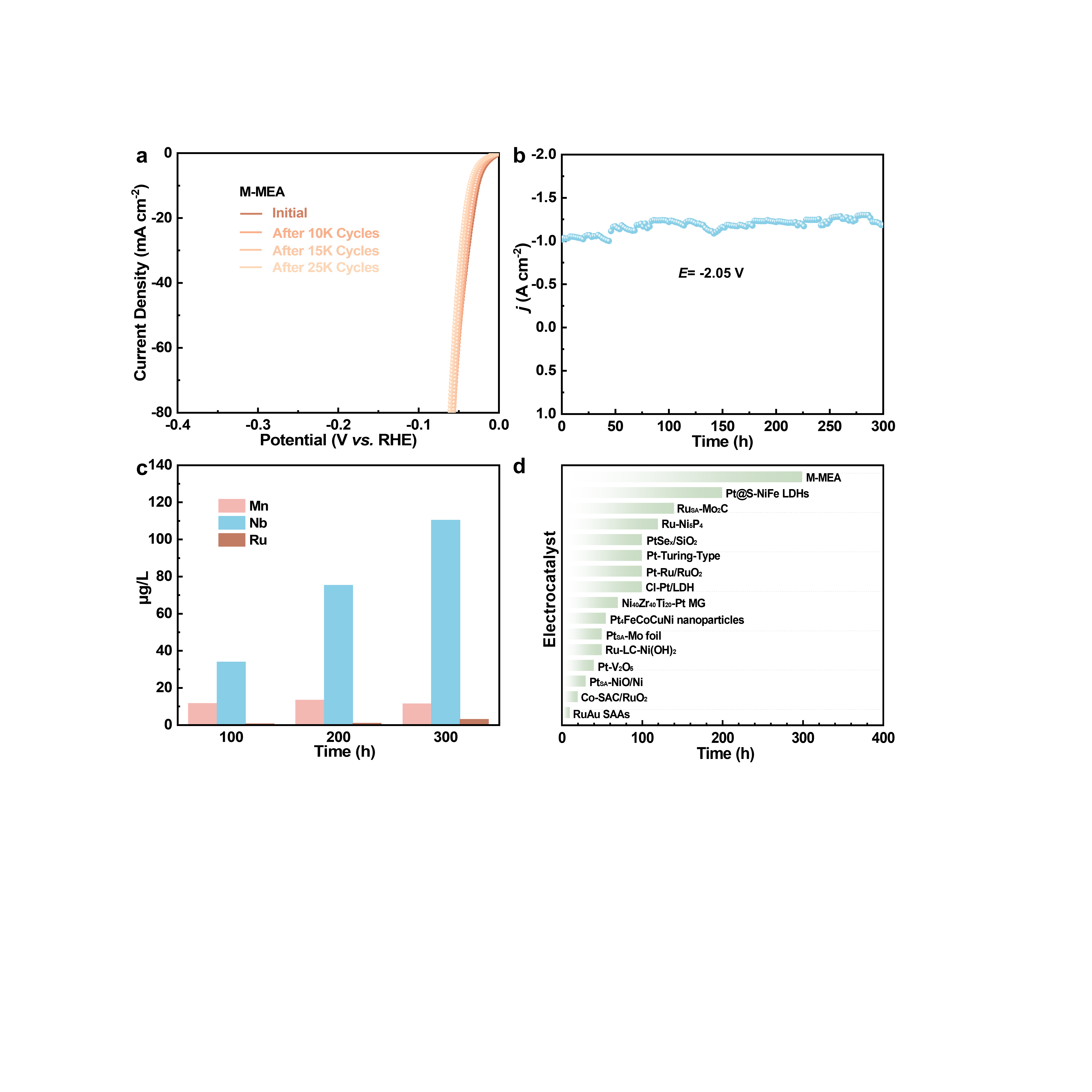


**Supplementary Fig. 15.** Stability test of M-MEA film. The LSV curves after 25,000 cycles accelerated durability test (ADT) for M-MEA film (a). (b) The chronoamperometry measurement of the M-MEA film at the potential of –2.05V (1.2 A cm^-2^) for 300 hours . (c) The variation of Ru, Mn and Nb concentration in the electrolyte with working time at a current density of 1.2 A cm^-2^. (d) Comparison among different noble alloy catalyst.

**
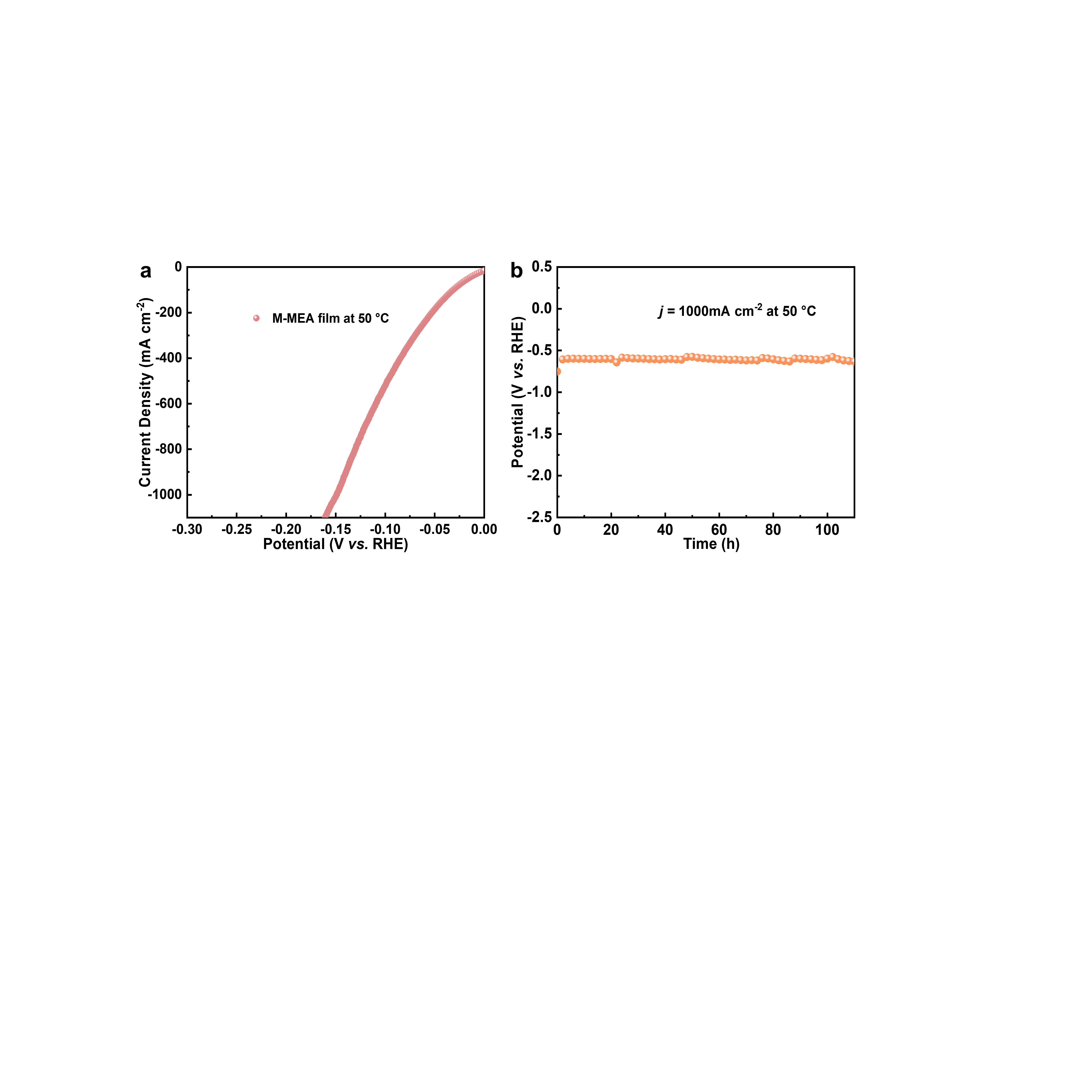
**

**Supplementary Fig. 16.** Industrial application of M-MEA film. (a) LSV curve at a scan rate of 5 mV s^-1^ (with iR compensation) at 50 °C. (b) Stability test of M-MEA film (without iR compensation) at 50 °C in 1 M KOH solution at -1000 mA cm^-2^.


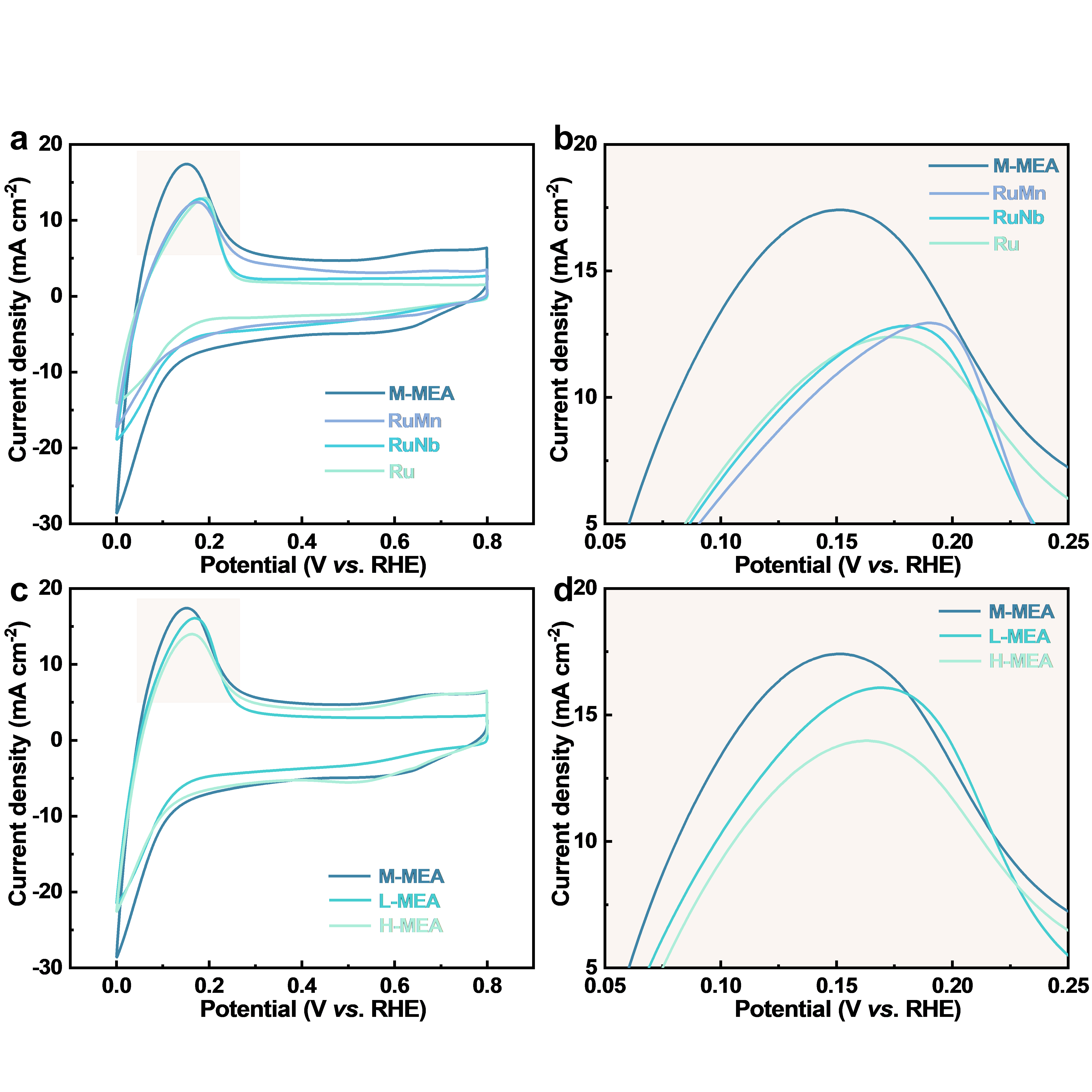


**Supplementary Fig. 17.** Underpotentially deposited hydrogen (HUPD) peak analysis. (a, c) CV curves of M-MEA film and comparative samples measured in 1.0 M KOH at a scan rate of 50 mV/s. (b, d) Enlarged CV curves of gray area in (a, c).


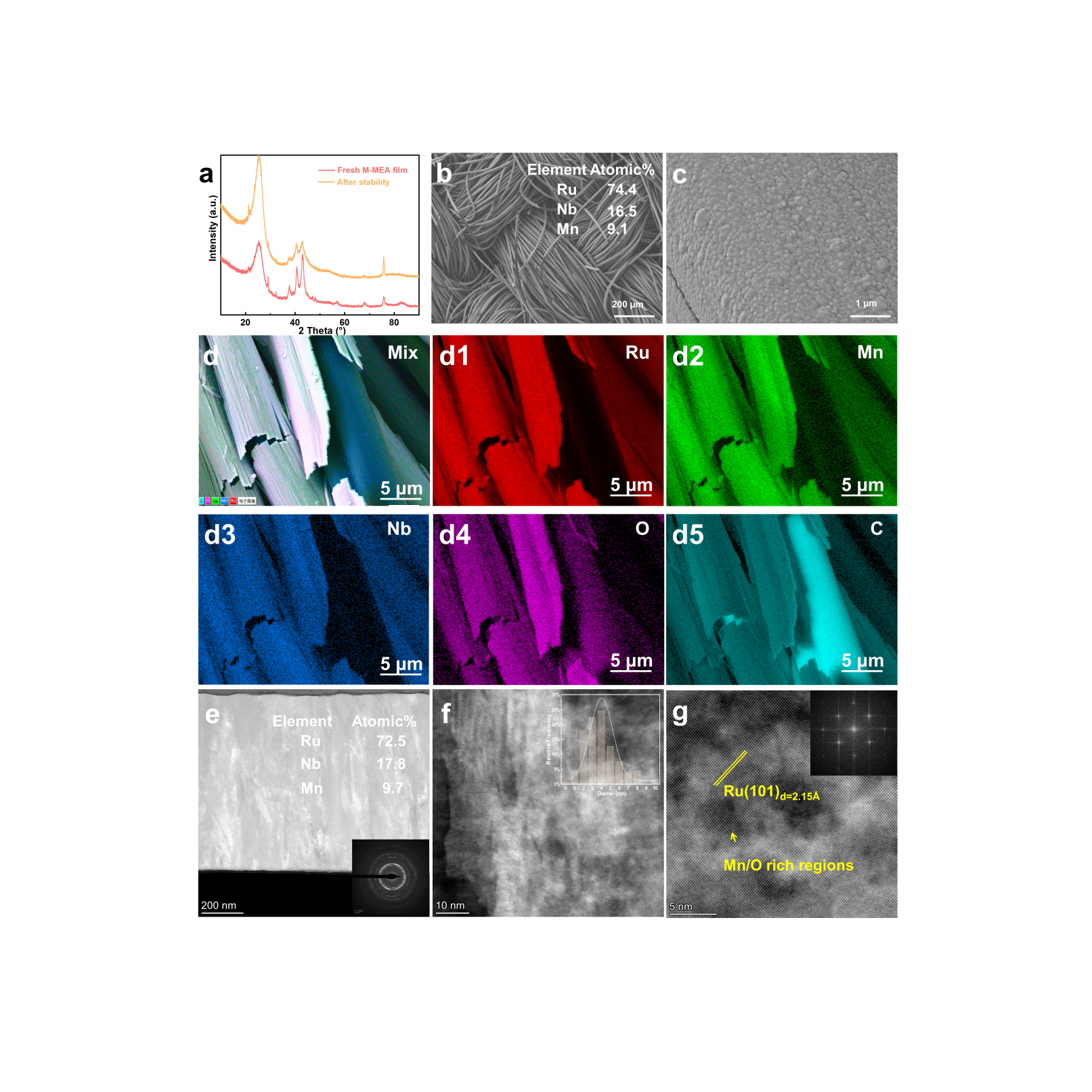


**Supplementary Fig. 18.** The structure of M-MEA film after stability. (a) The XRD patterns of the M-MEA film after electrolysis. (b–d5) SEM and EDS characterization of the M-MEA film after electrolysis. TEM images and statistical chart of Mn rich phase size after electrolysis (e–g).

**
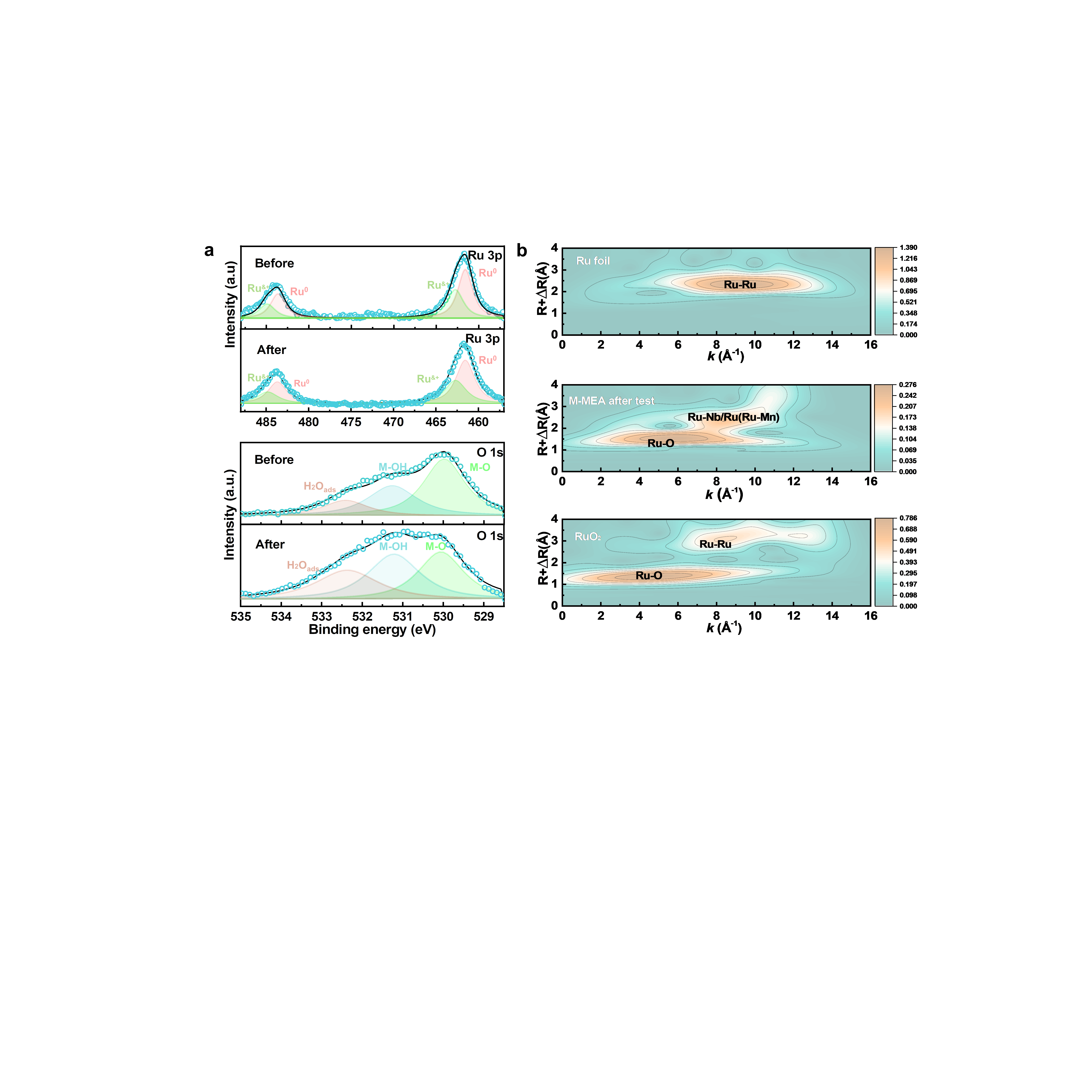
**

**Supplementary Fig. 19.** The valence of M-MEA film after stability. (a) XPS characterization of the M-MEA film before and after electrolysis. (b) The wavelet transform of the Ru K-edge EXAFS after tested sample. While the coordination environment of the film after testing differs slightly from that of the fresh film, there is no notable alteration, implying that the primary structures of the two samples remain mostly unchanged.

**
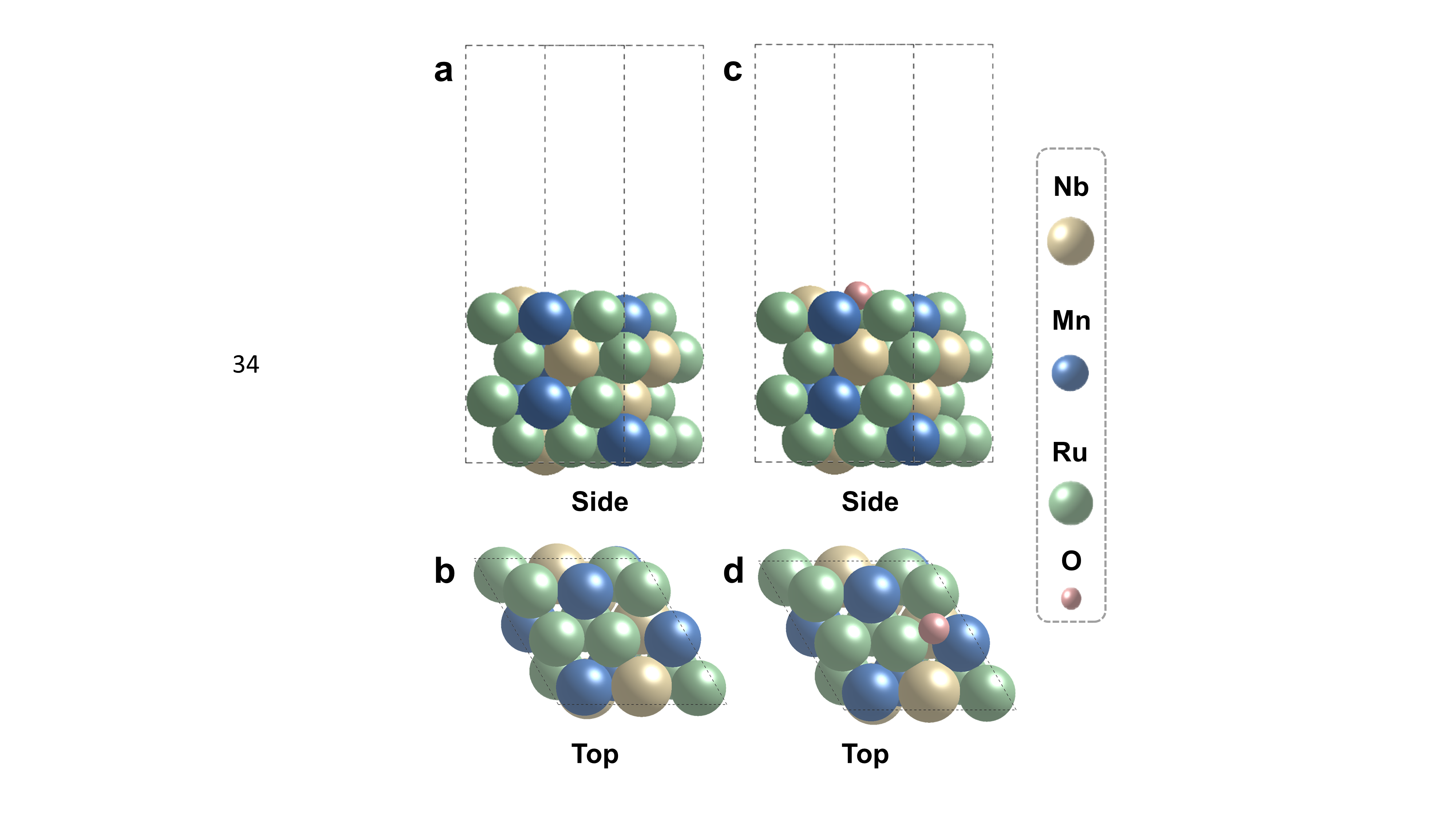
**

**Supplementary Fig. 20.** DFT simulation of atomic configuration: the side (a) and top (b) for without O model. the side (c) and top (d) for with O model.

**
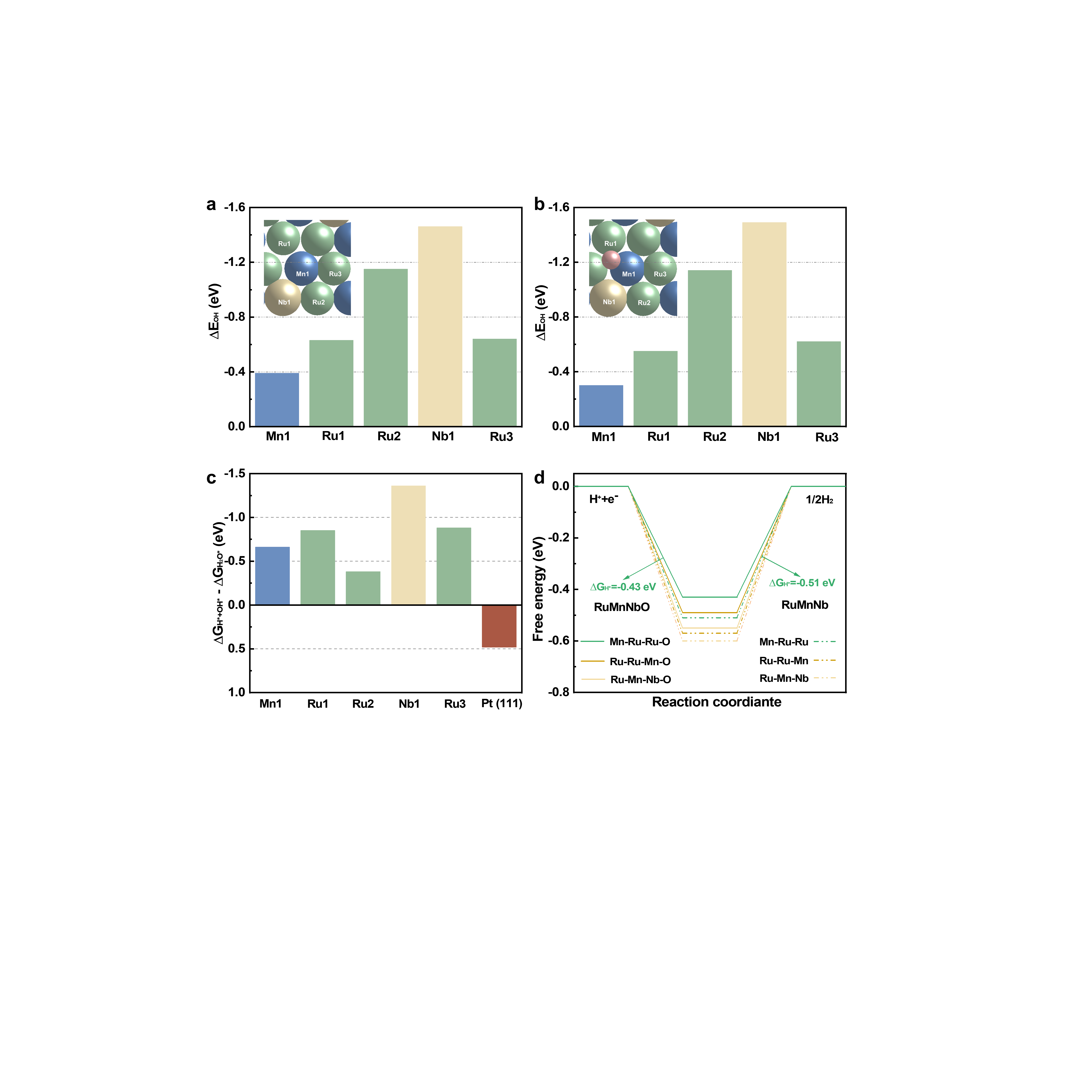
**

**Supplementary Fig. 21.** Adsorption energy of OH on Ru, Mn and Nb sites of the two models. (a) without O model. (b) with O model. (c) Corresponding energy change during the dissociation of H2O* to H* and OH* on Ru, Mn and Nb sites and Pt(111). (d) Gibbs free energy (∆GH*) profiles on tripleatom sites at the surfaces of the with and without O models. The insets are the corresponding atomic configurations after H* adsorption onto Mn-Ru-Ru-O and Mn-Ru-Ru sites of two model.


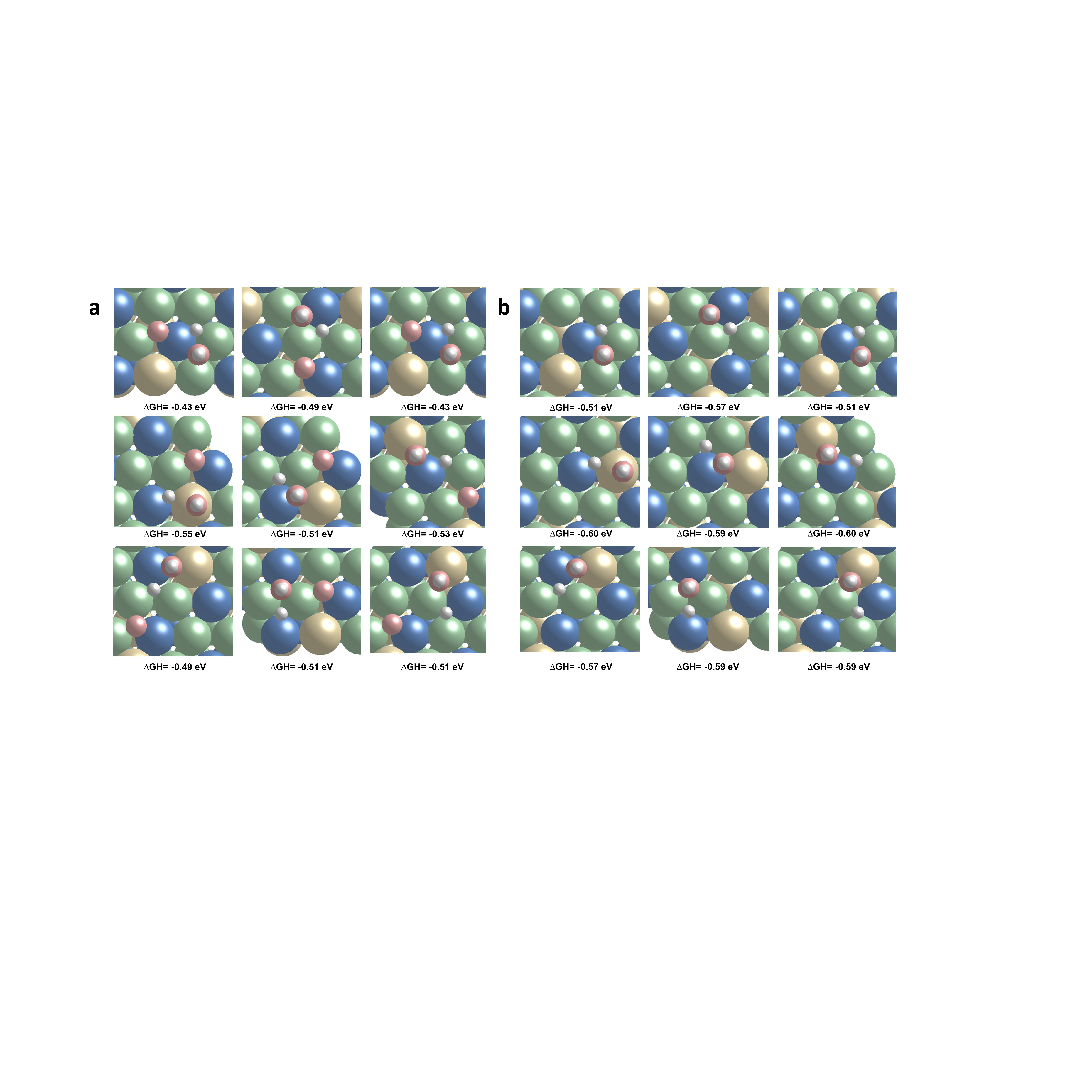


**Supplementary Fig. 22.** The representative atomic configurations with corresponding ∆GH* after H* and OH* adsorption at the triple-atom sites of (a) the with O models and (b) the without O models.

**Supplementary Table 1.** Economic cost for fabrication of M-MEA film by using raw materials.

| Raw material | Price per unit (g) | Quantity (mg) | Total price |
| --- | --- | --- | --- |
| Ru | $\$$12.7 | 0.7 | $\$$0.00889 |
| Mn | $\$$0.002 | 0.07 | - |
| Nb | $\$$0.09 | 0.2 | - |
| Total | / | / | $\$$0.00889 |

Note: The mass of H_2_ produced by M-MEA film operating at a current density of 1.2 A/cm^2^ for 300 hours can be calculated from the amount of transferred charge. Integrating the i-t curve yields the amount of transferred charge (*Q*=1.274*10^6^ C). The cost of the film is $\$$0.00889. Assuming that all the transferred charges are used to generate hydrogen gas, the mass of hydrogen (*m_H_*) is 1.32*10^-2^ kg. The cost per kilogram (*C*) of hydrogen is:

$$C=\frac{\$0.00889}{m_{H}}=0.67 \$/kg$$

**Supplementary Table 2.** Summary of the Ru k-edge XANES spectra and average valent state of Ru for as-dep and after stability tested M-MEA films.

| Sample | Energy(eV) | Valence of Ru |
| --- | --- | --- |
| RuO_2_ | 22117.3 | 4 |
| M-MEA | 22118.7 | 1.3 |
| M-MEA after tested | 22118.9 | 1.5 |
| Ru foil | 22121.6 | 0 |

**Supplementary Table 3.** EXAFS fitting parameters at the Ru *k*-edge for various samples (*Ѕ_0_^2^*=0.939).

| Sample | Shell | *C*N*^a^* | *R*(Å)*^b^* | *σ*2 (Å2)*^c^* | ΔE0(eV)*^d^* | *R* factor |
| --- | --- | --- | --- | --- | --- | --- |
| Ru foil | Ru–Ru | 12.0* | 2.676 | 0.0043 | -5.5 | 0.0067 |
| M-MEA | Ru–O | 3.7 | 2.055 | 0.0093 | 0.2 | 0.0101 |
|  | Ru–Mn | 2.8 | 2.513 | 0.0033 | 2.9 |  |
|  | Ru–Nb/Ru | 8.6 | 2.712 |  |  |  |
| M-MEA after tested | Ru–O | 3.9 | 2.032 | 0.0081 | -3.5 | 0.0212 |
|  | Ru–Mn | 3.1 | 2.555 | 0.0045 | 5.1 |  |
|  | Ru–Nb/Ru | 7.8 | 2.716 |  |  |  |
| RuO_2_ | Ru–O | 6.2 | 1.971 | 0.0030 | -0.9 | 0.0170 |
|  | Ru–Ru | 2.1 | 3.105 | 0.0028 | -4.6 |  |
|  | Ru–Ru | 7.7 | 3.538 |  |  |  |

*^a^CN*, coordination number; *^b^R*, the distance between absorber and backscatter atoms; *^c^σ*^2^, the Debye Waller factor value; *^d^ΔE*_0_, inner potential correction to account for the difference in the inner potential between the sample and the reference compound; *R* factor indicates the goodness of the fit. *S*_0_^2^ was fixed to 0.939, according to the experimental EXAFS fit of Ru foil by fixing *CN* as the known crystallographic value. * This value was fixed during EXAFS fitting, based on the known structure of Ru. fitting space: R space; *k*-weight = 3. A reasonable range of EXAFS fitting parameters: 0.800 < *Ѕ*_0_^2^ < 1.000; *CN >* 0; *σ*^2^ > 0 Å^2^; |Δ*E*_0_| < 15 eV.

**Supplementary Table 4.** Summary of the overpotential at 10 mA/cm^2^ for M-MEA films and comparison sample.

| Sample | M-MEA | L-MEA | H-MEA | RuNb | RuMn | Ru | commercial Pt/C | Pt foil |
| --- | --- | --- | --- | --- | --- | --- | --- | --- |
| Overpotential/mV | 18 | 20.3 | 25.3 | 26.1 | 30.4 | 29.6 | 23.4 | 213 |

**Supplementary Table 5.** Summary of the Solution resistance and Charge transfer resistance for M-MEA films and comparison sample.

| Samples | Solution resistance (ohm) | Charge transfer resistance (ohm) |
| --- | --- | --- |
| M-MEA | 1.68 | 1.48 |
| H-MEA | 1.96 | 2.05 |
| L-MEA | 2.53 | 1.96 |
| RuNb | 1.81 | 2.36 |
| RuMn | 1.57 | 2.56 |
| Ru | 1.44 | 2.87 |
| Pt/C | 1.48 | 3.30 |

**Supplementary Table 6.** The comparison of overpotential (η) at 10 mA/cm^2^ and Tafel slope of the M-MEA film with recently reported electrocatalysts in 1.0 M KOH.

| Catalysts | η@10 mA/cm^2^ | Tafel slope | References |
| --- | --- | --- | --- |
| M-MEA film | 18 | 21.6 | this work |
| RuMn NSBs-250 | 20 | 32.2 | [36] |
| Ru@Cr-FeMOF | 21 | 26 | [37] |
| RuP_2_(RP-CPM) | 21 | 47.3 | [38] |
| IrMo_0.59_ alloy | 23 | 60 | [39] |
| RuAu SAAs | 24 | 37 | [40] |
| Pt_SA_-NiO/Ni | 26 | 27 | [41] |
| Ru/NC | 26 | 36 | [42] |
| (FeCoNiB_0.75_)_97_Pt_3_ HEMG | 27 | 31 | [43] |
| IrP_2_@NC | 27 | 50 | [44] |
| IrNi@OC-430 | 28 | 50 | [45] |
| Pt_SA_/MoSe_2_ | 29 | 41 | [46] |
| Ru-MoO_2_ | 29 | 44 | [47] |
| Pt_SA_/Mo foil | 31 | 31.1 | [48] |
| PdPtCuNiP HEMG | 32 | 37.4 | [49] |
| Ru/N-doped C | 32 | 53 | [50] |
| Ni_40_Zr_40_Ti_20_-Pt_3_ MG | 37 | 30 | [51] |
| Ru/NC-400 | 39 | 49 | [52] |
| Ni_3_N/Ru/NCAC | 42 | 59 | [53] |
| Co_SA_/RuO_2_ | 45 | 58 | [54] |
| RuSiW | 46 | 49 | [55] |

**Supplementary Table 7.** The comparison of TOF of the M-MEA film with recently reported electrocatalysts in 1.0 M KOH at 50mV (*vs.*RHE).

| Catalysts | TOF (s^-1^) | References |
| --- | --- | --- |
| Ru-2D Mo | 0.27 | [58] |
| H-RuSe_2_ | 0.34 | [59] |
| Pt_3_Fe/(NMCS-A) | 1.32 | [60] |
| Ir_25_Ni_33_Ta_42_ MG | 1.76 | [61] |
| Rh/RhOx | 2 | [62] |
| Ru_SA_-Mo_2_C | 3.16 | [63] |
| Ru/Co@OG | 6.2 | [64] |
| PdPtCuNiP HEMG | 6.58 | [49] |
| Pt-Fe_5_Ni_4_S_8_ | 9.87 | [65] |
| IrMo alloy | 15.4 | [66] |
| Al_73_Mn_7_Ru_20_ | 24.3 | [23] |
| Ru/NC-400 | 32 | [52] |
| M-MEA film | 34.8 | this work |

**Supplementary Table 8.** The comparison of stability time of the M-MEA film with recently reported electrocatalysts in 1.0 M KOH at different current densities.

| Catalysts | Time/h | Current/mA | references |
| --- | --- | --- | --- |
| RuAu SAAs | 10 | 10 | [40] |
| Co_SA_/RuO_2_ | 20 | 10 | [54] |
| Pt_SA_-NiO/Ni | 30 | 20 | [41] |
| Pt-V_2_O_5_ | 40 | 10 | [67] |
| Ru-LC-Ni(OH)_2_ | 50 | 1000 | [31] |
| Pt_SA_-Mo foil | 50 | 900 | [48] |
| Pt_4_FeCoCuNi nanoparticles | 55 | 10 | [68] |
| Ni_40_Zr_40_Ti_20_-Pt_3_ MG | 70 | 10 | [51] |
| Cl-Pt/LDH | 100 | 50 | [69] |
| Pt-Ru/RuO_2_ | 100 | 250 | [7] |
| Pt-Turing-Type | 100 | 500 | [70] |
| PtSe_x_/SiO_2_ | 100 | 140 | [71] |
| (Ni_5_P_4_-Ru) | 120 | 35 | [72] |
| Ru_SA_-Mo_2_C | 140 | 100 | [63] |
| Pt@S-NiFe LDHs | 200 | 100 | [73] |

**Supplementary Table 9.** ICP-OES results (μg/L) of the electrolyte after HER stability test for different time at current densities of 1.2 A/cm^2^.

| Time | Ru | Mn | Nb |
| --- | --- | --- | --- |
| 100h | 0.8 | 11.7 | 34.0 |
| 200h | 1.0 | 13.5 | 75.3 |
| 300h | 3.1 | 11.4 | 110.4 |

**Supplementary Table 10.** The OH adsorption sites and adsorption energies (∆GOH) in two models.

| ∆GOH/eV | Mn1 | Ru1 | Ru2 | Nb1 | Ru3 |
| --- | --- | --- | --- | --- | --- |
| With O model | -0.30 | -0.55 | -1.14 | -1.49 | -0.62 |
| Without O model | -0.39 | -0.63 | -1.15 | -1.46 | -0.64 |

**Supplementary Table 11.** The H2O dissociation sites and dissociation energy (∆GD) in O embedding model.

| ∆GD/eV | Mn1 | Ru1 | Ru2 | Nb1 | Ru3 | Pt (111) |
| --- | --- | --- | --- | --- | --- | --- |
| With O model | -0.66 | -0.85 | -0.38 | -1.36 | -0.88 | 0.48 |
